# PHABULOSA Controls the Quiescent Center-Independent Root Meristem Activities in *Arabidopsis thaliana*

**DOI:** 10.1371/journal.pgen.1004973

**Published:** 2015-03-02

**Authors:** Jose Sebastian, Kook Hui Ryu, Jing Zhou, Danuše Tarkowská, Petr Tarkowski, Young-Hee Cho, Sang-Dong Yoo, Eun-Sol Kim, Ji-Young Lee

**Affiliations:** 1 Boyce Thompson Institute for Plant Research, Ithaca, New York, United States of America; 2 School of Biological Sciences, Seoul National University, Seoul, Korea; 3 Laboratory of Growth Regulators, Faculty of Science, Palacky University and Institute of Experimental Botany AS CR, Olomouc, Czech Republic; 4 Department of Protein Biochemistry and Proteomics, Centre of the Region Hana for Biotechnological and Agricultural Research, Faculty of Science, Palacky University, Olomouc, Czech Republic,; 5 School of Life Sciences and Biotechnology, Korea University, Seoul, Korea; Peking University, CHINA

## Abstract

Plant growth depends on stem cell niches in meristems. In the root apical meristem, the quiescent center (QC) cells form a niche together with the surrounding stem cells. Stem cells produce daughter cells that are displaced into a transit-amplifying (TA) domain of the root meristem. TA cells divide several times to provide cells for growth. SHORTROOT (SHR) and SCARECROW (SCR) are key regulators of the stem cell niche. Cytokinin controls TA cell activities in a dose-dependent manner. Although the regulatory programs in each compartment of the root meristem have been identified, it is still unclear how they coordinate one another. Here, we investigate how PHABULOSA (PHB), under the posttranscriptional control of SHR and SCR, regulates TA cell activities. The root meristem and growth defects in *shr* or *scr* mutants were significantly recovered in the *shr phb* or *scr phb* double mutant, respectively. This rescue in root growth occurs in the absence of a QC. Conversely, when the modified *PHB*, which is highly resistant to microRNA, was expressed throughout the stele of the wild-type root meristem, root growth became very similar to that observed in the *shr*; however, the identity of the QC was unaffected. Interestingly, a moderate increase in *PHB* resulted in a root meristem phenotype similar to that observed following the application of high levels of cytokinin. Our protoplast assay and transgenic approach using *ARR10* suggest that the depletion of TA cells by high PHB in the stele occurs via the repression of B-ARR activities. This regulatory mechanism seems to help to maintain the cytokinin homeostasis in the meristem. Taken together, our study suggests that PHB can dynamically regulate TA cell activities in a QC-independent manner, and that the SHR-PHB pathway enables a robust root growth system by coordinating the stem cell niche and TA domain.

## Author Summary

Plant roots are programmed to grow continuously into the soil, searching for nutrients and water. The iterative process of cell division, elongation, and differentiation contributes to root growth. The quiescent center (QC) is known to maintain the root meristem, and thus ensure root growth. In this study, we report a novel aspect of root growth regulation controlled independently of the QC by PHABULOSA (PHB). In *shr* mutant plants, PHB, which in the meristem is actively restricted to the central region of the stele by SHORTROOT (SHR) via *miR165/6*, suppresses root meristem activity leading to root growth arrest. A high concentration of PHB in the stele does this by modulating B-ARR activity through a QC-independent pathway. Accordingly, we observed a significant recovery of root meristem activity and growth in the *shr phb* double mutant, while the QC remained absent. However, the presence of QC may be required to sustain continuous root growth. On the basis of our results, we propose that SHR maintains root growth via two separate pathways: by modulating PHB levels in the root stele, and by maintaining the QC identity.

## Introduction

Plants, unlike animals, grow continuously and dynamically adjust their architecture. Meristems at the apices of shoots and roots harbor stem cells and serve as the center of cell division and growth. In the root apical meristem (RAM), the quiescent center (QC) maintains a stem cell population to form a stem cell niche [1], [2]. There are two main pools of stem cells, the proximal and distal stem cells, which are named based on their position relative to the QC (reviewed by [3]). Distal stem cells produce the root cap. Post-embryonic root growth in an apical direction, hereafter referred to as root growth, occurs via iterative divisions of the transit-amplifying cells (TA cells), derived from the proximal stem cells, and their subsequent cell elongation.

Two main regulatory programs are crucial for the root stem cell niche: one directed by PLETHORAs (PLTs) and the other by SHR and SCR. The PLTs play an essential role in establishing the QC and the RAM during embryogenesis [4]. SHR and SCR, the GRAS family transcription factors, act together to maintain the stem cell niche and root growth [5], [6], [7]. When *SHR* or *SCR* is knocked out, QC cells are not maintained and root growth terminates prematurely. The essential role of SHR and SCR in QC specification was further evident in a regeneration study of QC cells. In contrast to wild-type plants, QC cells failed to recover in *shr* and *scr* mutants after laser ablation [8].

The role of the QC within the stem cell niche has been extensively studied in *Arabidopsis*. Laser ablation of QC cells first indicated that the QC maintains the adjacent stem cell population [2]. In this study, the role of the QC in distal stem cell maintenance is evident; however, the extent to which the QC contributes to the maintenance of the proximal stem cells and the TA cell population is unclear [3]. In *wox5* mutants, which affects the identity and morphology of the QC cells, the size of the TA cell population is unaltered and root growth is relatively normal even though the distal stem cells are not maintained [9]. Furthermore, root regeneration experiments have suggested that the QC is not required for the regeneration competency of the meristem cells [10].

In addition to the stem cell niche, cell division activities of the TA cell population significantly affect root growth. Cytokinin signaling plays a key role in this process. In the absence of cytokinin, TA cell proliferation is strongly inhibited. As a result, severe defects in root growth are observed in the triple mutant of *ARR1, 10*, and *12*, the type B *Arabidopsis* response regulator (B-ARR) genes, and the triple mutant of *AHK2, 3* and *4*, cytokinin receptor genes [11], [12]. Under high cytokinin levels, the transition of TA cell status from dividing to differentiating is promoted, reducing the size of the meristem and slowing down root growth [13]. This process is mediated by ARR1 and 12, which up-regulate the expression of *SHY2*, an inhibitor of auxin signaling [14]. A recent study indicated that SCR in the QC regulates this process in a non-cell autonomous manner [15].

The *HOMEODOMAIN-LEUCINE ZIPPER Class III* (*HD-ZIP III*) genes are key regulators of shoot and root meristems (reviewed by [16], [17]). In *Arabidopsis*, the *HD-ZIP III* family consists of five members: *PHABULOSA* (*PHB*), *PHAVOLUTA* (*PHV*), *CORONA* (*CNA*), *REVOLUTA* (*REV*), and *ARABIDOPSIS THALIANA HOMEOBOX8* (*ATHB8*) [18]. These genes are post-transcriptionally regulated by microRNA (*miRNA*) *165/6* [19], [20]. Studies have shown that during root meristem establishment, HD-ZIP III proteins are excluded from the basal embryo pole [21], [22]. *SERRATE* (*SE*), a zinc-finger (ZnF) domain-containing protein, is a component of dicing bodies that process pre-miRNAs together with DICER-LIKE1 (DCL1) [23]. In *se* mutants, the production of mature miRNAs is reduced, thus *PHB* and *PHV* expand to the basal embryo pole, and formation of the root meristem fails [21]. SHR and SCR also suppress *HD-ZIP III* in the root by directly regulating the transcription of *miR165A* and *166B* [24]. However, unlike *SE, SHR* and *SCR* mainly affect post-embryonic root development.

In summary, significant progress has been made to identify key transcription factors and signaling molecules in the function and maintenance of the root stem cell niche and TA cells. However, it remains unclear how these factors and molecules are integrated to control root growth. Here, we show that PHB, the concentration of which is controlled by SHR, governs TA cell activities by repressing B-ARRs. Such a repressive effect of PHB on B-ARR activities is enhanced by high cytokinin. Therefore, restricting the PHB level via the SHR-microRNA pathway is a critical step for maintaining root growth.

## Results

### Elevated PHB levels suppress root proximal meristem activity

**Fig 1:**
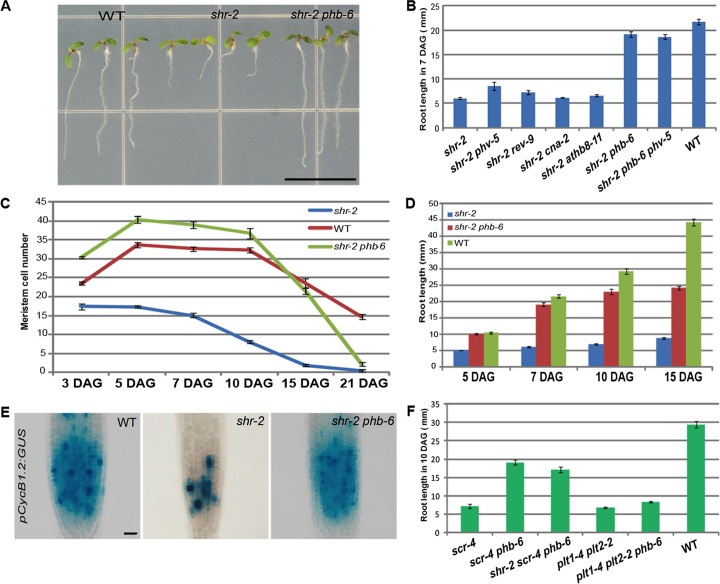
PHB regulates root meristem activity downstream of SHR-SCR. (**A**) Seedlings of *shr phb* (7 DAG) mutants display significant recovery in root length. (**B**) Growth restoration in *shr phb* roots compared with other *shr HD-ZIP III* mutants and *shr phb phv-5* mutants. (**C**) Meristem size over time in the *shr, shr phb*, and wild-type roots. (**D**) Root length measured over a time course. The *shr phb* roots grow in a more determinate manner than do the wild-type roots. (**E**) Expression of *pCycB1.2:GUS* in the *shr, shr phb*, and wild-type roots (5 DAG). (**F**) *phb*-mediated recovery in root meristem/growth is specific to the SHR-SCR pathway. The error bars represent the standard error (n = 20–40 plants). Scale bars: A, 1 cm; E, 20 μm.

Previously, we reported that SHR-dependent expression of *miR165/6* in the ground tissue layer quantitatively and spatially restricts *HD-ZIP III* expression within the stele, which is necessary for normal xylem tissue patterning [24]. We also observed that the short root phenotype of *shr* mutants could be partially recovered when *miR165/6* was miss-expressed in the ground tissue or stele, suggesting that ectopic *HD-ZIP III* expression reduced root growth in this mutant. However, at the time, it was not known which of the *HD-ZIP III* genes was responsible for root growth. We tested this by analyzing double mutants of *shr* and each of the five *HD-ZIP III* genes. In the double mutant of *shr* and *phb-6* (hereafter *phb*), a loss-of-function mutant allele of *PHB* with a Ds insertion, the roots were significantly longer than the *shr* mutant roots and more or less comparable to the wild type 7 days after germination (DAG) (Fig. 1A; S1A Fig.). Under the growth conditions used in this experiment, *phb* single mutant roots were slightly shorter than the wild type (S1A Fig.), which excluded the possibility that the rescue of root length in *shr phb* mutants is an additive effect. The negative influence of *PHB* on root length in the *shr* mutant was further supported in our suppressor screen of EMS-mutagenized *shr* plants. In this screen, we identified two new loss-of-function *phb* alleles, which are *phb-15* and *phb-16* (S1A Fig.). Mutations in *CORONA* (*CNA*) or *ATHB8*, two other *HD-ZIP III* genes, however, failed to rescue root length in the *shr* mutant, whereas *PHAVOLUTA* (*PHV*) and *REVOLUTA* (*REV*) elicited only a weak recovery (Fig. 1B). Introducing a knockout mutant of *PHV*, the HD-ZIP III family member most closely related to *PHB* [19], to the *shr phb* did not yield a further increase in *shr phb* root length (Fig. 1B).

To understand how root length is rescued in *shr phb* mutants, we analyzed root growth and meristem activities in comparison with the wild type and *shr* mutant (Fig. 1C-1E). The *shr* mutant roots barely grew beyond 5 DAG, whereas *shr phb* roots displayed growth similar to, or faster than, wild-type roots until 7 DAG. After 7 DAG, however, the growth of these plants decelerated and had almost ceased by 15 DAG (Fig. 1D). Consistent with the recovery of root growth, the meristem in the *shr phb* roots was significantly larger than the *shr* meristem (S1B Fig.). A time-course analysis of root meristem [25] showed that both wild-type and *shr phb* roots displayed an increase in meristem size between 3 and 5 DAG, whereas *shr* roots showed a steady decrease (Fig. 1C). At 5 DAG, the meristem of *shr phb* roots was larger than that of the wild type (Student’s *t*-test; *P* < 0.001, α = 0.05). We also examined meristem cell division using a G_2_-M cell cycle marker, *pCycB1.2:GUS* [26]. GUS expression at 5 DAG indicated full recovery of cell division in the proximal meristem of the *shr phb* roots (Fig. 1E). Interestingly, *pCycB1.2:GUS* expression was comparable in the wild-type and *shr phb* roots at 15 DAG, despite the deceleration of root growth in the *shr phb* plants (S1C and 1D Fig.). These data suggest that the slowing of growth is not caused by a complete loss of cell cycle activity but rather it may be due to some other defects that are not directly related to meristematic function. For example, the *shr phb* mutant has defective development of the phloem sieve elements, which could affect the transport of nutrients required for root growth (S2A Fig.).

SCR regulates *miR165/6* expression together with SHR [24]. Thus, we examined whether *PHB* also affects root growth activity controlled by SCR, and indeed, *scr-4 phb* roots displayed a significant recovery in the size of the proximal meristem (S1B Fig.) and root length compared with *scr-4* roots (Fig. 1F). Consistent with these findings, *shr scr-4 phb* triple mutant plants also recovered root length (Fig. 1F). Finally, we investigated whether *phb* could suppress the short root phenotype of *plt1 plt2* mutants. However, *plt1-4 plt2-2 phb* triple mutants did not exhibit any noticeable recovery in root length (Fig. 1F). Taken together, these data suggest that PHB is a key downstream factor of SHR and SCR in the regulation of the meristem and root growth.

### TA cell proliferation and root growth in the absence of QC

**Fig 2:**
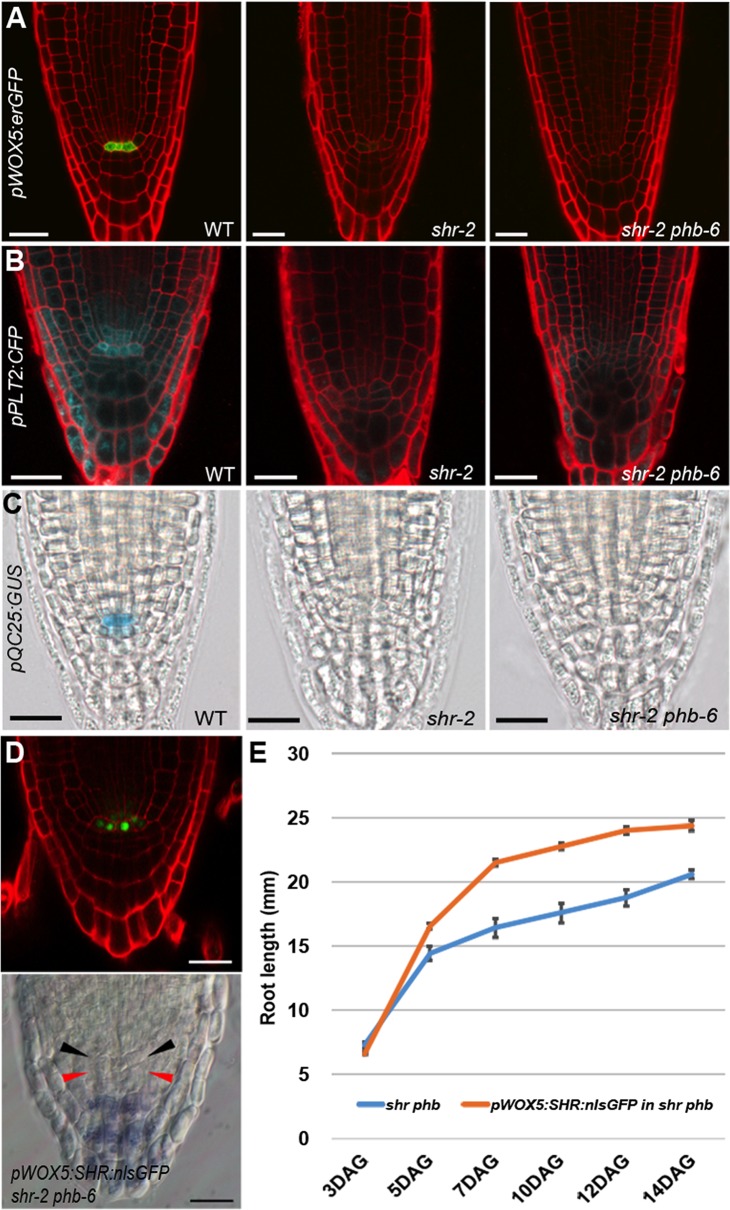
Recovery of root meristem/growth activity in the absence of a functional QC. (**A–C**) Expression analysis of QC markers in the wild-type, *shr*, and *shr phb* roots (5 DAG). (**A**) *pWOX5:erGFP*. (**B**) *pPLT2:CFP*. (**C**) *pQC25:GUS*. (**D**) Analysis of QC status in a *shr phb* root expressing *pWOX5:SHR:nlsGFP*, by confocal microscopy (upper panel) and Lugol staining (lower panel). Black arrowhead indicates the QC where SHR-nlsGFP is observed in the upper panel. Red arrowhead indicates the recovered columella stem cell layer where starch granules are absent. **(E)** The comparison of root length between *shr-2 phb-6* and *pWOX5:SHR:GFP;shr-2 phb-6*. Root lengths were measured from seedlings 3–14 DAG. Scale bars: **A–C**, 25 μm; **D**, 20 μm. The error bars represent the standard error (n = 5–10 plants).

The short root phenotype of the *shr* and *scr* mutants has been ascribed to the failure of stem cell maintenance in the absence of a QC. The lack of stem cell maintenance in *shr* mutants is supported by a steady decrease in root meristem size upon germination (Fig. 1C). Because *shr phb* mutants displayed both an active increase in root meristem size and a rescue in root length, we examined whether the QC is rescued in the *shr phb* roots. To our surprise, none of the QC markers analyzed in actively growing *shr phb* roots indicated QC recovery (Fig. 2; S2B Fig.). The expression of *pWOX5:erGFP* (endoplasmic reticulum-localized GFP) and *pQC25:GUS*, which is strong and specific to the QC cells in wild-type roots, was below the level of detection in the QC regions of both *shr phb* and *shr* roots (Fig. 2A and 2C) [9], [27]. In the wild-type roots, expression of *pPLT2:CFP, pPLT1:CFP, pSCR:erGFP, pAGL42:erGFP*, and p*QC6:erGFP* [6], [28], [29], [30], are at their highest in the QC and are lower in the neighboring cell types. Analysis of these markers also indicated the lack of a QC in the *shr phb* roots (Fig. 2B; S2B Fig.). We also analyzed *pWOX5:erGFP* in the wild-type, *shr* and *shr phb* embryos to determine whether the different *WOX5* expression patterns in the *shr* and *shr phb* embryos could explain the recovery of root growth. Up to the mid-torpedo stage, we detected *GFP* expression in both wild-type and *shr* embryos (S3A Fig.). Thereafter, contrary to that in the wild-type embryos, *pWOX5:erGFP* expression declined dramatically in the *shr* mutants and was no longer detectable (S3B Fig.). The dynamics of *pWOX5:erGFP* expression in the *shr phb* mutant were indistinguishable from those in the *shr* mutant.

Without the QC, the distal stem cells cannot be maintained. Lugol staining indicated that distal stem cells differentiate prematurely in *shr phb* roots, similar to that in *shr* roots visualized by the presence of starch granules that marks differentiated columella cells (S3C Fig.). This is consistent with the lack of QC marker expression in the *shr phb* mutant, which supports the observation that the QC is absent in these plants.

Recently, it was suggested that the QC promotes root growth by regulating the transition between cell proliferation and differentiation in a non-cell autonomous manner [15]. Therefore, we asked whether the presence of the QC in the *shr phb* mutant could fully restore root growth to wild-type levels. To investigate this, SHR fused to nuclear-localized GFP was expressed in *shr phb* roots under the *WOX5* promoter (*pWOX5:SHR:nlsGFP; shr phb*). Unlike the *shr phb* roots in which *WOX5* expression diminished in the late stage of embryogenesis, three independent *shr phb* transgenic lines maintained SHR-nlsGFP expression in the QC region of post-embryonic roots, suggesting at least a partial recovery of QC driven by WOX5 (Fig. 2D). Furthermore, the meristem of the *shr phb* roots expressing *WOX5* appeared to be more organized than that of the *shr phb* plants, and the columella stem cells were restored as indicated by Lugol staining (Fig. 2D). Expression of *WOX5* in the QC position increased the growth potential of the *shr phb* roots; however, their lengths were significantly shorter than those of the wild-type roots (Fig. 2E). Together these data suggest that proliferation of TA cells is controlled by the level of PHB, and that the PHB level plays a significant role in root growth, in combination with the QC maintenance and other unknown factors that affect root growth.

### PHB regulates TA cell proliferation and root growth from the stele

**Fig 3:**
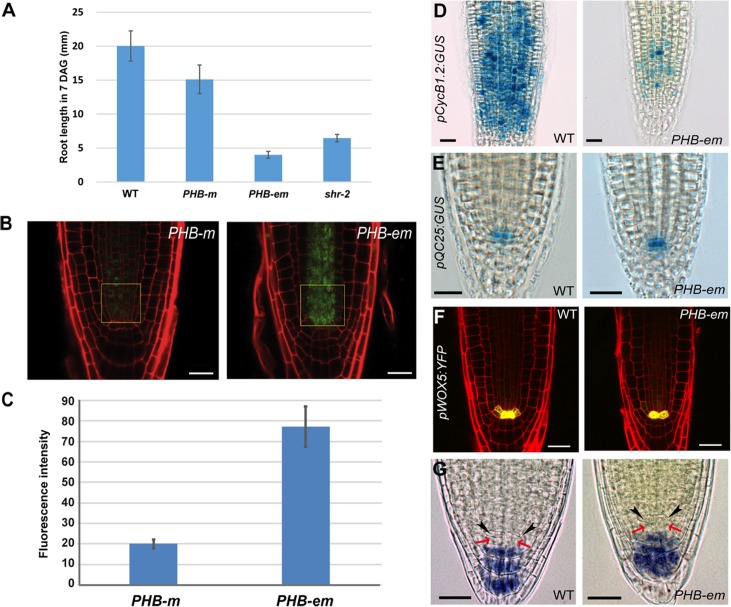
PHB in the stele regulates root meristem and growth activity in a QC-independent manner. (**A)** A comparison of root lengths in wild-type, *pWOL:PHB-m:GFP_NLS_*, p*WOL:PHB-em:GFP_NLS_* and *shr-2* plants (7 DAG). The error bars represent the standard error (n = 9–14 plants). (**B, C**) Quantitative comparison of PHB-GFP levels in the root stele cells expressing *pWOL:PHB-m:GFP*_*NLS*_ and *pWOL:PHB-em:GFP*_*NLS*_. Fluorescence intensity was measured for GFP in the boxed area of the panel (**B**). Error bars represent standard error (n = 11) (**D**) *pCycB1.2:GUS* expression shows drastic reduction in cell division potential in the proximal meristem. Expression of *pQC25:GUS* (**E**) and *pWOX5:YFP* (**F**) in the *PHB-em* roots. (**G**) Starch granule accumulation as visualized by Lugol’s staining in the *pWOL:PHB-em:GFP*_*NLS*_ roots. The black arrowheads and red arrows indicate the QC and columella stem cells, respectively. Scale bars: B, 20 μm; D-G, 25 μm.

SHR posttranscriptionally restricts *HD-ZIP III* mRNA to the center of the stele [24]. In the root meristem of *shr* mutants, PHB is observed throughout the stele (S4B Fig.), which results in the short root phenotype that can be rescued by the misexpression of *miR165/6* in the ground tissue. Hence, high PHB levels in the stele of the root meristem probably influence root growth. To confirm this, we increased the dosage of PHB in a specific manner throughout the stele in the wild-type root meristem. We modified *PHB* so that it was not efficiently targeted for degradation by *miR165/6*, and then expressed it under the *WOODEN LEG* (*WOL*) promoter, which drives gene expression in stele cells in the root meristem [31] (S5 Fig.). Two types of modified PHB were used: one with a single silent mutation that partially interferes with *miR165/6* binding (*PHB-m*) [24], and the other with four silent mutations that strongly block *miR165/6* binding (*PHB-em*) (S4A Fig.). Driving *PHB-m* under the *PHB* promoter results in the broadening of PHB domain throughout the stele as we previously reported (S4B Fig.). Expression analysis using confocal microscopy showed that PHB-GFP is present at a significantly higher level in the stele cells of roots expressing *pWOL:PHB-em:GFP*_*NLS*_ than in those expressing *pWOL:PHB-m:GFP*_*NLS*_ (Fig. 3B and 3C). Consistent with high PHB-GFP levels inhibiting normal root growth, roots expressing *pWOL:PHB-em:GFP*_*NLS*_ were much shorter than those expressing *pWOL:PHB-m:GFP*_*NLS*_, and were either similar to or shorter than the *shr* mutant roots (Fig. 3A). Accordingly, the meristem size of the *pWOL:PHB-em:GFP*_*NLS*_ roots was also dramatically reduced (S4C Fig., upper panel). Furthermore, these root meristems had significantly fewer stele cells compared to wild-type root meristems (S4C Fig., lower panel). TA cell proliferation, as detected by *pCycB1.2:GUS* expression, decreased dramatically in these roots compared to the wild-type ones (Fig. 3D). Collectively, these data suggest that high levels of PHB in the stele cells of the root meristem suppress TA cell proliferation and root growth, and therefore signaling from the stele is a rate-limiting factor in root growth.

Next, we examined the QC status in the *pWOL:PHB-em:GFP*_*NLS*_ lines that displayed root meristem and growth phenotypes similar to that of the *shr* mutant. The expression patterns of *pQC25:GUS* and *pWOX5:YFP* in *pWOL:PHB-em:GFP*_*NLS*_ lines were similar to that in the wild type (Fig. 3E and 3F); their expression persisted even in older roots (15 DAG; S4D and S4E Fig.). Lugol staining of roots at 5 and 7 DAG also supported the presence of a QC (Fig. 3G). Nevertheless, in the *pWOL:PHB-em:GFP*_*NLS*_ roots, the cells in the QC position were often enlarged and showed aberrant cell divisions (S4F Fig.). This phenotype was more frequent in older roots (7 DAG or older). Furthermore, *in situ* analysis indicated that there is a decline in *WOX5* mRNA levels in these transgenic roots (S4G Fig.). These data further confirm that PHB regulation of the TA cell proliferation and root growth is independent of the QC status.

### A genome-wide survey of downstream pathways of PHB in the root stele

To determine how high levels of PHB in the stele suppress root growth in *shr* mutant plants, we generated stele-specific genome-wide gene expression data from wild-type, *shr-2*, and *shr-2 phb-6* roots. In this experiment, we used a protoplast/cell-sorting technique to isolate and collect the stele cells from roots expressing *pWOL:erGFP* in three genotypes, and mRNAs in the stele were profiled using the *Arabidopsis* Tiling 1.0R arrays from Affymetrix [32] (S5 and S6A Fig.; S1 Table). To identify genes or pathways that are regulated by PHB, we identified genes that are differentially expressed between the *shr* mutant and the wild type (Corrected *p*-value, FDR <0.05; fold difference >2). Under the given criteria, 1933 genes were revealed as differentially expressed between the two (S2 Table). We then investigated the expression patterns of these genes in the *shr, shr phb*, and wild-type plants using principal component analysis (PCA) and QT clustering [33] (S6B and S6C Fig.; S2 Table). These analyses indicated that most changes in gene expression in the *shr* mutant (92% represented by 1781 genes in clusters 1–3) were due to high levels of PHB. In total, 1170 genes were activated (cluster 1) and 611 genes were repressed (clusters 2 and 3) by PHB in *shr* mutant plants (S6C Fig.).

**Fig 4:**
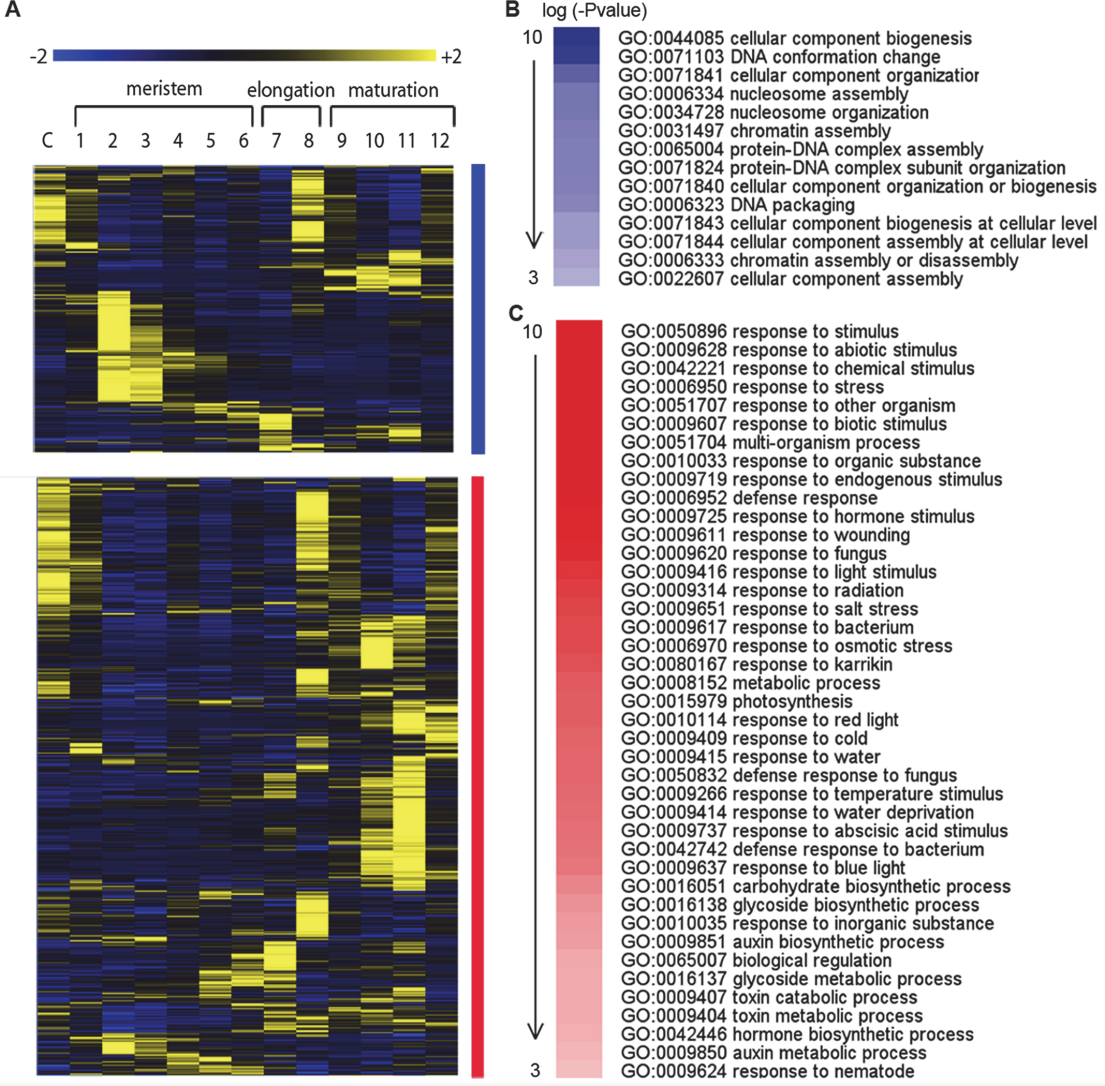
Downstream genes of SHR-PHB in the root stele. (**A**) Hierarchical clustering of expression in the *Arabidopsis* root along the longitudinal axis for genes that are repressed (blue; genes in cluster 2 and 3 in S6C Fig.) or activated (red; genes in cluster 1 in S6C Fig.) by a high level of PHB in *shr* mutants. Expression values are normalized by row. (**B**) Over-represented biological functions of genes that are repressed (*P*-value marked in blue) or (**C**) activated (*P*-value marked in red) by a high level of PHB in *shr* mutants.

Next, we analyzed the spatial expression of genes that are activated or repressed by high levels of PHB in the *shr* mutant using the expression data generated following micro-dissection of a root along the longitudinal axis and the cell type-specific root expression data (Fig. 4A; S7 Fig.) [24], [29], [34], [35], [36]. Hierarchical clustering of genes present in the root micro-dissection data indicates that ∼40% of the genes repressed by PHB are highly expressed in the TA domain, whereas those activated by PHB are enriched in the maturation zone. GO term enrichment analysis suggested that the pathways repressed by high levels of PHB are related to cell proliferation processes [37] (S3 Table; Fig. 4B). This suggests that in the stele, PHB actively represses genes involved in TA cell activities. In contrast, pathways activated by PHB are biotic and abiotic stress responses, and those involved in hormone production and signaling. This includes genes in auxin, ABA, and cytokinin biosynthesis and response (S3 Table; Fig. 4B). Cell-type specific expression of nearly 40% of genes repressed by PHB is enriched in the xylem precursor cells (S7 Fig.) [38]. This is consistent with the recovery of xylem patterning in *shr phb* mutant plants [24]. In contrast to the genes repressed by PHB, those activated by PHB showed enriched expression in multiple cell types. The overrepresentation of genes involved in multiple signaling processes might explain the lack of enriched patterns of gene activation in response to PHB in particular cell types.

A balance between auxin and cytokinin signaling is important for root growth and meristem activity [39]. Our stele-specific mRNA analysis suggests that the SHR-PHB pathway might affect the balance of these two hormones. We further examined this by analyzing the expression pattern of *pDR5:YFP*_*venus*_ and *pPIN1:PIN1:GFP*, in *shr, shr phb*, and wild-type roots (S8A–S8C Fig.). As reported recently [40], *pPIN1:PIN1:GFP* expression declined in both 5- and 10-day-old *shr* roots. Its expression was moderately restored in the roots of *shr phb* plants, which is consistent with our data from stele profiling. A similar expression pattern was observed for *pDR5:YFP*_*venus*_. These results suggest that high PHB levels in *shr* mutant plants reduce auxin signaling (transport/biosynthesis) in the root meristem, but its effect appears to be relatively minor.

### PHB locally regulates genes involved in cytokinin biosynthesis in the root meristem

Previous studies have suggested the existence of a homeostatic feedback loop mechanism between auxin and cytokinin in the root, in which cytokinin positively regulates a subset of genes involved in auxin biosynthesis [41], [42]. Our tissue-specific stele expression profiling revealed that high PHB levels in *shr* mutants up-regulate genes involved in auxin biosynthesis/metabolism. In addition, the level of PIN1 protein, which is degraded by high levels of cytokinin, was reduced in the *shr* mutant and was then moderately restored in the *shr phb* mutant (S8B and S8C Fig.) [43]. Moreover, cytokinin levels are increased in *shr* roots [44]. These results led us to examine whether high PHB in *shr* mutants affects the cytokinin status in the roots.

**Fig 5:**
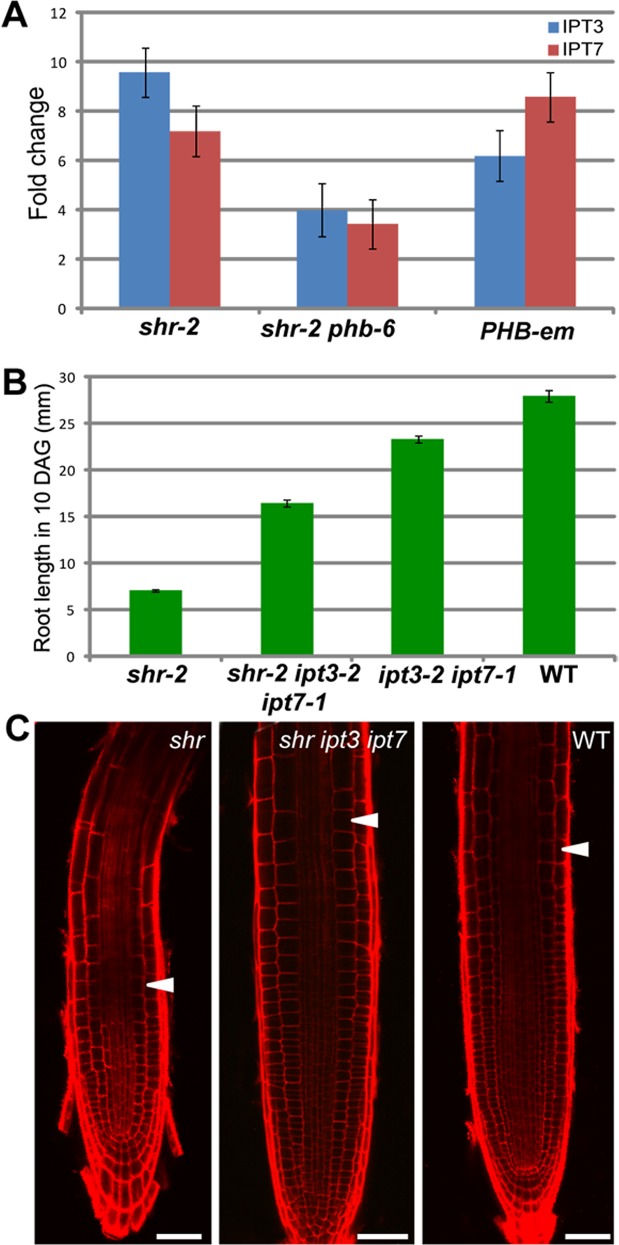
The SHR-PHB pathway controls root growth in a cytokinin-dependent manner (A) Relative mRNA levels of *IPT3* and *IPT7* in *shr, shr phb*, and *pWOL:PHB-em:GFP*_*NLS*_ (*PHB-em*) roots. Data are normalized to Expression levels in wild-type roots. (**B**) Root lengths (10 DAG) and (**C**) meristem images (5 DAG) in *shr, shr ipt3-2 ipt7-1* mutants. The error bars represent the standard error (n = 20 plants). Scale bar: 50 μm. The white arrowhead marks the end of the meristem.

*ISOPENTENYL TRANSFERASE* (*IPT*) *1, 3, 5*, and *7* promote cytokinin biosynthesis in *Arabidopsis* roots [45]. Our stele profiling data indicated that *IPT5* and *7* are upregulated by PHB in the *shr* stele. Previous expression data in the *shr* and wild-type root tips revealed the up-regulation of *IPT3* and *7* in *shr* mutants [35]. We corroborated these findings by quantifying the expression levels of *IPT3, 5*, and *7* in the wild-type, *shr*, and *shr phb* roots using qRT-PCR. Consistently, mRNA levels of *IPT3* and *7* were found to increase in *shr* roots compared with wild-type roots, and their levels were partially restored in *shr phb* roots (Fig. 5A). These results suggest that high levels of PHB enhance the expression of *IPT3* and *7*. We further confirmed this by measuring the mRNA levels of *IPT3* and *7* in the root tips of transgenic plants expressing *pWOL:PHB-em:GFP*_*NLS*_ (Fig. 5A).

We then asked whether PHB regulation of *IPT3* and *7* influences root meristem/growth activity by measuring the size of the root meristem and the length of the root in *shr ipt3-2 ipt7-1* triple mutant plants. The *shr ipt3-2 ipt7-1* plants showed a recovery in the root length and meristem size compared to *shr* plants, but to a much lesser degree than the *shr phb* roots (Fig. 5B and 5C; *P* < 0.001, α = 0.05). Under our growth conditions, the *ipt3-2 ipt7-1* double mutant roots were slightly shorter than those of wild-type plants. Collectively, these data indicated that PHB influences root meristem and growth activity in the *shr* plants, at least partly via the regulation of cytokinin biosynthesis.

### PHB in the *shr* mutant interferes with B-ARR activities

**Fig 6:**
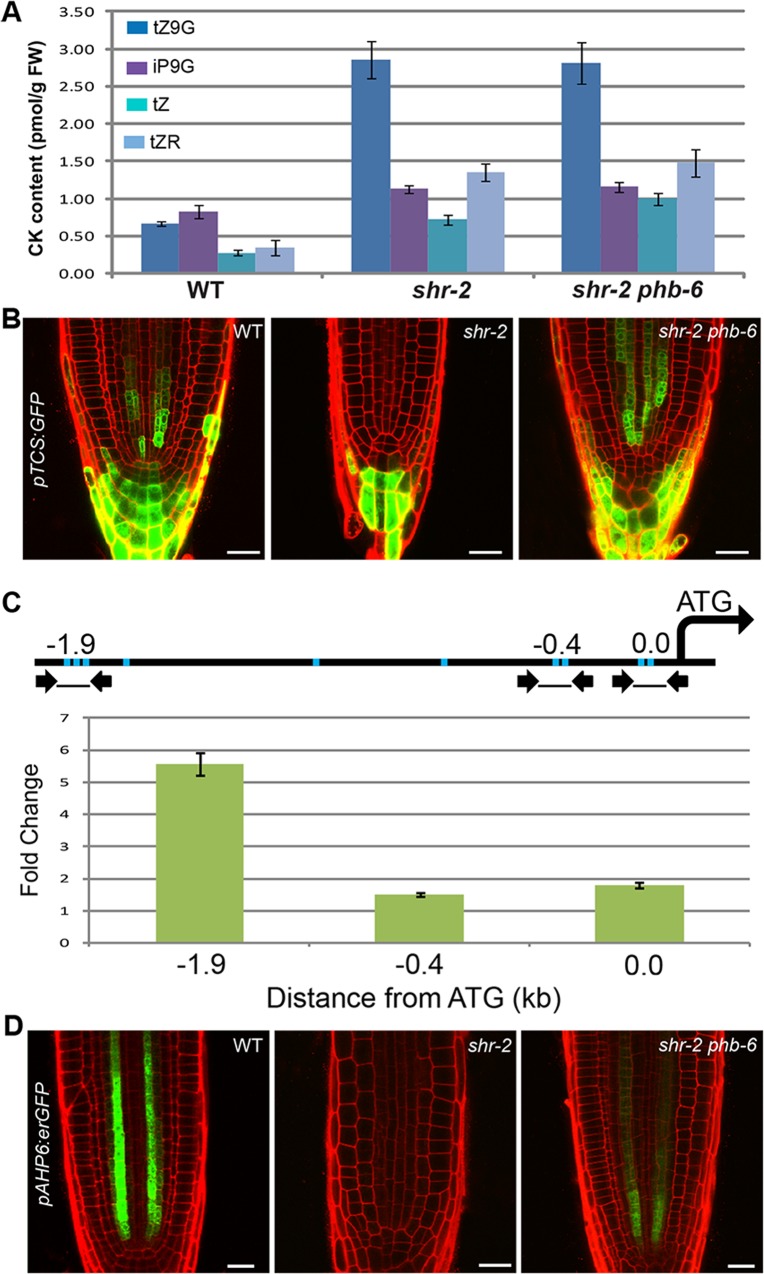
The SHR-PHB pathway and cytokinin signaling. (**A**) Cytokinin (CK) expression in wild-type, *shr*, and *shr phb* roots. tZ, Transzeatin; tZR, tZ Riboside; iP9G, N^6^-(Δ^2^-isopentenyl) adenine-9-glucoside; tZ9G, tZ-9-glucoside. (**B**) Expression of *pTCS:GFP* in wild-type, *shr*, and *shr phb* roots. (C) Real-time PCR and ChIP showing PHB binding to the ARR7 promoter. Line diagram represents the *ARR7* promoter region. Arrows and blue bars indicate primers and B-ARR binding elements (GGATT/AATCT), respectively. (**D**) Recovery in *pAHP6:erGFP* expression in the *shr phb* roots in comparison with *shr* roots. The error bars represent the standard deviation (A) and standard error (C) (n = 3 biological replicates). Scale bars: 25 μm. CK, cytokinin.

To determine the significance of cytokinin biosynthesis regulation by PHB in the root meristem, we measured cytokinin levels in wild-type, *shr*, and *shr phb* roots. Consistent with the previous study, an increase in cytokinin level was observed in the *shr* roots (Fig. 6A; S9A Fig.). Various forms of trans-zeatin increased by approximately four-fold in *shr* plants in comparison to the wild type. However, we failed to detect a decline in global cytokinin levels in *shr phb* roots, as compared with those in *shr* roots. This suggests that there is another layer of regulation mediated by PHB in the cytokinin pathway, which is probably involved in restoring the *shr phb* root growth.

In our stele profiling data, the expression of multiple *A-ARRs*, such as *ARR5, 6, 7, 8, 9*, and *15*, was slightly induced in *shr* mutants but was further upregulated in *shr phb* mutants (S9B Fig.). We validated this finding by qRT-PCR analysis (S9C Fig.). Considering that global cytokinin levels were comparable in both *shr* and *shr phb* roots, this result implied that high PHB levels in the *shr* mutant might be actively suppressing the expression of *A-ARR*s.

All *A-ARR* genes have cis-regulatory elements that are directly bound by B-ARRs [46]. *pTCS:erGFP* is a synthetic cytokinin reporter construct with B-ARR binding elements, and its expression therefore reflects the transcriptional activity of B-ARRs. When the *in vivo* cytokinin level increases in the wild-type root meristem, more B-ARRs are activated and thereby enhance *pTCS:erGFP* expression [47]. Thus, we investigated the involvement of PHB in B-ARR function by analyzing the expression of *pTCS:erGFP* in the wild-type, *shr*, and *shr phb* roots [47]. Despite a high cytokinin level, *shr* roots displayed a dramatic reduction in *pTCS:erGFP* expression (Fig. 6B). In contrast, *TCS* expression was restored in *shr phb* mutants to levels higher than those in wild-type plants. Thus, high levels of PHB appear to interfere with B-ARRs that induce *TCS* transcription in the root stele. Consistent with this theory, *TCS* expression was found to be absent or very weak in the root stele of *phb-7d* mutants carrying a semi-dominant point mutation in the microRNA target site of *PHB* [24] (S9E Fig.).

A high level of PHB could interfere with B-ARR activity directly or indirectly. To determine how PHB affects B-ARR activity, we developed a transgenic line expressing *PHB-em* fused to the glucocorticoid receptor (GR) under the control of the *PHB* promoter, and crossed a line expressing this construct with *pTCS:erGFP* (S10A and S10B Fig.). The resulting F_1_ seedlings were treated with dexamethasone for 3 hours and *pTCS:erGFP* expression in roots was analyzed in comparison with that of the untreated control plants. GFP expression in roots harboring both *pTCS:erGFP* and *pPHB:PHB-em:GR* was much weaker than that of roots carrying *pTCS:erGFP* alone, suggesting that the PHB-em:GR is leaky. Nevertheless, with dexamethasone treatment, we found a significant reduction in GFP expression in roots with both *pTCS:erGFP* and *pPHB:PHB-em:GR*, not in roots with *pTCS:erGFP* alone. Since the response of *pTCS:erGFP* occurs only after 3 hours, we investigated the possibility that PHB expressed at high levels can interact with the promoters of *A-ARR* genes. *ARR7*, an *A*-*ARR* that is highly responsive to PHB in the root meristem, was selected and used to perform a chromatin immunoprecipitation (ChIP) assay (Fig. 6C; S9B and S9C Fig.). The ChIP assay, combined with quantitative real-time PCR, indicated enrichment of PHB binding to the *ARR7* promoter region where B-ARR binding elements are present. We then tested whether an enhanced *ARR7* expression contributes to root length recovery in *shr phb* mutants by analyzing root length in *shr phb arr7* triple mutant plants (S9D Fig.). The *shr phb arr7* roots showed a partial reduction in root length compared with that of *shr phb*, suggesting that A-ARRs may influence the restoration of root growth in *shr phb* mutant plants (Student’s *t*-test; *P* < 0.001, α = 0.05).

The suppression of negative regulators of cytokinin signaling was recently shown to enhance cytokinin sensitivity in *Arabidopsis* roots [48]. Consistent with this, our expression analysis of two cytokinin-sensitive markers, *AHP6* [49] and *S32* [30], [44], indicated the enhanced cytokinin sensitivity in the *shr*. The expression of *pAHP6:erGFP*, which is inhibited by high levels of cytokinin, was absent in *shr* mutants and was partially restored in *shr phb* roots (Fig. 6D). The expression of *pS32:erGFP*, which is induced by high levels of cytokinin, increased and expanded in the *shr* roots but was consistent with the wild-type pattern in *shr phb* roots (S9F Fig.). Our stele-specific microarray data also supported the dynamics of these markers. Thus, it appears that the increased cytokinin sensitivity in *shr* mutants is due to the suppression of *A-ARR* expression by PHB.

### PHB in the *shr* suppresses root growth by blocking B-ARR activity

**Fig 7:**
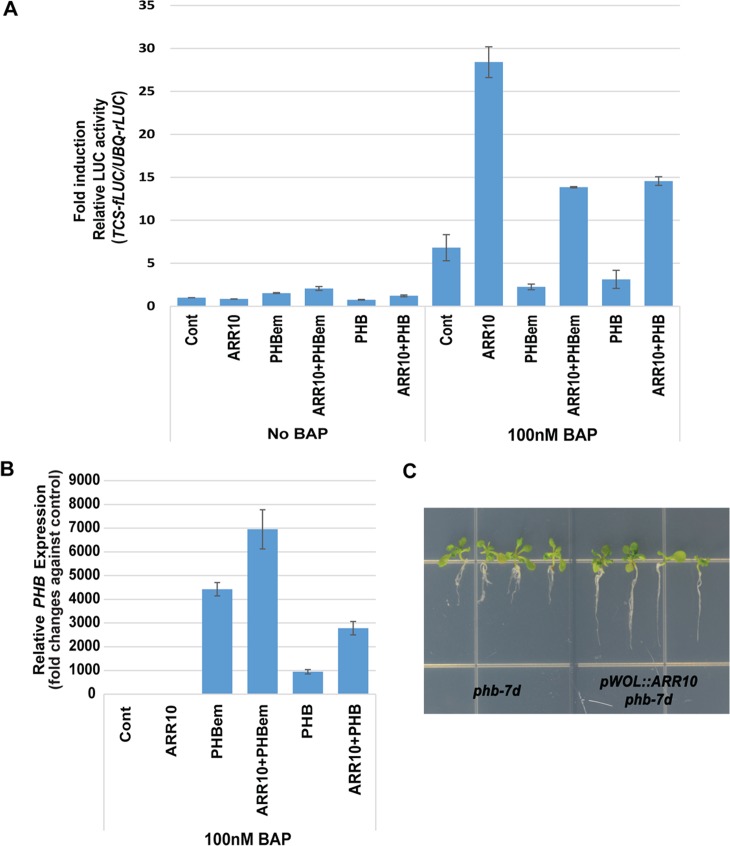
PHB suppresses B-ARR activities in the presence of high cytokinin. A protoplast assay that measures the ARR10 activities in the presence of PHB. **(A)** A high dosage of PHB (*p35S:PHB-em* and *p35S:PHB*) suppressed ARR10 activities under the high cytokinin. For the reporter assay, *pUBQ:rLUC* was co-transfected as an internal transfection control. Relative TCS activities with effectors (fold induction) were inferred by measuring the ratios between luminescence from fire fly luciferase (*pTCS:fLUC*) and luminescence from renilla luciferase (*pUBQ:rLUC*) and then dividing those values with the ratio obtained from control without an effector and BAP treatment. The error bar represents standard deviation. (**B**) *PHB* expression from 100 nM BAP-treated protoplasts is analyzed by real-time RT-PCR. Relative *PHB* expression was obtained by measuring fold changes against *PHB* expression in the control protoplasts. The error bar represents standard deviation (n = 3). (**C**) Increasing ARR10 levels by expressing it under *WOL* promoter restores the root growth in the *phb-7D*. BAP, 6-benzylaminopurine.

Our data so far indicate that high levels of PHB inhibit B-ARR activity. Since ChIP data suggest that PHB acts on B-ARR binding targets, we hypothesized that PHB might block B-ARRs in a quantifiable manner. To test this hypothesis, we employed a protoplast transient assay system (Fig. 7A; S11A Fig.). We measured luciferase activity driven by the TCS promoter (*pTCS:LUC*) in protoplasts transfected with 1) *ARR10* alone, 2) *PHB-em* alone, 3) *ARR10* and *PHB-em*, 4) *PHB* alone, and 5) *ARR10* and *PHB* which were all driven by the *35S* promoter. In two independent experiments, *PHB* mRNA levels expressed from *p35S:PHB-em* were found higher than *PHB* from *p35S:PHB* (Fig. 7B; S11B Fig.). In the absence of exogenous cytokinin, we did not detect a dramatic difference in luciferase activity between the protoplasts transfected with different constructs. However, in the presence of 6-benzylaminopurine (BAP), PHB protein expressed by *p35S:PHB-em* strongly suppressed ARR10 activity. This repressive effect was also observed in protoplasts transfected with *PHB-em* alone. However, the effect of PHB expressed by *p35S:PHB* on ARR10 activities was inconsistent. This seems to be due to the influence of *miRNA 165/6* that targets *PHB* mRNA but not *PHB-em* (Fig. 7B; S11B Fig.). Collectively, these data confirm that high levels of PHB repress B-ARR activities, more effectively under high cytokinin.

Our protoplast assay further indicated that the root growth defect in *shr-2, phb-7d* and *pWOL:PHB-em:GFP*_*NLS*_ plants might be due to depleted, as opposed to enhanced, cytokinin signaling. If this mechanism were important in regulating root growth, supplying more B-ARR to the *shr* or *phb-7d* mutant would overcome the inhibitory effect of PHB on root growth. To test this hypothesis, we introduced *pWOL:ARR10* into the *phb-7d* mutant. Analysis of four T2 plants showed a noticeable restoration in root growth, supporting our hypothesis (Fig. 7C).

## Discussion

### PHB in the root stele regulates TA cell proliferation

Controlling the spatial domains/levels of *HD-ZIP III* genes is critical for multiple developmental processes: ad/abaxial organization of lateral organs [50], specification of apical/basal axis in embryos [22], and vascular patterning [24], [51]. In this study, we demonstrate that this regulation is also essential for proper organization of the root meristem and root growth. Among the five *HD-ZIP III* genes, *PHB* seems to be the major player, because deletion of the other *HD-ZIP III* genes did not restore root growth in the same way as *phb* did in the *shr* mutant. Consistent with the results of a previous study showing that SCR acts in conjunction with SHR to posttranscriptionally suppress *PHB* and other *HD-ZIP III*s, the *scr phb* mutant is also able to restore root length.

Despite recovery of root growth, we did not detect any visible sign (based on cell morphology and QC marker expression) of an accompanying recovery of QC cells in the *shr phb* mutant. TA cell proliferation becomes very active in *shr phb*, as indicated by the expression of *pCycB1.2:GUS*, and the size of the meristem over time, displayed very similar dynamics between wild-type and *shr phb* roots. These findings indicate that the proliferation of TA cells is prolonged in the absence of QC cells in *shr phb* mutants. Conversely, *pWOL:PHB-em:GFP*_*NLS*_ plants expressing a high level of PHB in the root stele cannot maintain TA cell proliferation and root growth while sustaining a QC. Therefore, PHB in the stele cells regulates TA cell activities required for root growth, independently of the QC in a non-cell autonomous manner. Interestingly, once the TA cells stop functioning in the *pWOL:PHB-em:GFP*_*NLS*_ roots, the QC starts to lose its identity as a secondary effect. In animal systems, the maintenance of stem cell niches requires a communication between stem cells and their differentiated progenies, or TA cells [52], [53], [54]. When TA cells in the intestine are damaged, mitotically inactive cells in the stem cell niche become active and show stem cell characteristics [55], [56]. These features suggest that the mechanisms of stem cell niche functioning in animals and plants might be similar, even though the components involved are distinct.

Root growth is a dynamic process. Growth accelerates to a certain time point and then decelerates to terminate (reviewed by [57]). During this process, the meristem structure also changes. Toward the end of the root growth period, the QC degenerates, which is defined by its cell division. This results in the transition of the closed meristem to an open meristem in the *Arabidopsis* root [58], [59]. Therefore, the presence of a QC appears to be important for maintaining root growth over time. In this context, the RAM might operate in a similar way to the shoot apical meristem, where the organizing center is important for prolonging the meristem activity [60]. *shr phb* roots have open meristems and grow in a determinate manner. This determinate root growth does not appear to be caused by the exhaustion of TA cells because the root meristem size of *shr phb* plants does not change from that of the wild type until 15 DAG. Therefore, the early termination of root growth in *shr phb* mutants might be due to the lack of QC cells. Consistent with this theory, when the QC is recovered in *shr phb* roots by expressing *pWOX5*::*SHR:nlsGFP*, the growth becomes more active and less determinate than the *shr phb*. However, the overall root growth in the presence of QC in these plants remained below the wild-type level. Together, these data suggest that there is another aspect of root growth regulation, or that the QC is not fully restored. It has been reported that root growth in *shr* mutants can be partially rescued via the expression of cell-autonomous SHR expression in the stele [24], [61]. Our further investigation suggests that this restores the defect in phloem development and accompanies a partial recovery in root growth (manuscript in preparation).

### PHB regulates root meristem/growth activity via alteration of B-ARR activities

Cytokinin affects root meristem and growth in a concentration-dependent manner [62]. Our study suggests that PHB regulates the activity of the root meristem and growth by two mechanisms: one involving cytokinin synthesis and the other involving changes in B-ARR activities. Our gene expression analysis indicated an up-regulation of *IPT3* and *IPT7* expression in accordance with elevated levels of *PHB*, and *vice versa*. Direct regulation of *IPT7* expression by PHB was recently demonstrated [63]. However, activation of this pathway is not sufficient to produce a change in global cytokinin levels in the root, as we could not detect a change in cytokinin levels in the *shr phb* roots compared to that in *shr* plants. Nevertheless, when we prevented the up-regulation of *IPT3* and *IPT7* by PHB in *shr* mutants by generating *shr ipt3 ipt7* triple mutants, root length and meristem size were partially restored. This is consistent with the results from our protoplast assay, which showed that the alteration of B-ARR activity by PHB is enhanced under high cytokinin levels.

Parallel to local cytokinin production, PHB regulates cytokinin signaling, and this process appears to be the main factor affecting root meristem and growth activity in the *shr* mutant. Despite the fact that cytokinin levels were similarly high in the *shr* and *shr phb* mutants, the expression of cytokinin-responsive markers was not consistent in *shr* and *shr phb* roots. Expression patterns of *pAHP6:GFP* and *pS32:GFP* indicated a higher cytokinin status in the *shr* root stele, while the *shr phb* mutants were similar to wild-type plants. On the other hand, *pTCS:GFP*, which reflects the transcriptional activities of B-ARRs is strongly suppressed in the *shr* and *phb-7d* mutants, but is recovered in the *shr phb* plants, demonstrating the effect of elevated PHB on cytokinin signaling pathway. Furthermore, consistent with the *pTCS:GFP* results, our stele-specific gene expression data obtained from the *shr, shr phb*, and wild-type roots indicated that a high level of PHB in the *shr* stele actively suppresses the expression of at least 50% of *A-ARR*s in the genome. On the basis of these data, together with the ChIP-PCR result suggesting that PHB can bind to the *ARR7* promoter, we propose that increased PHB expression suppresses the cytokinin-mediated transcriptional activation by *B-ARR*s. This hindrance in B-ARR activity on one hand seems to increase cytokinin sensitivity in the *shr* roots, but on the other hand it affects TA cell proliferation leading to root growth arrest in a manner comparable to a cytokinin depleted mutant such as the *arr1 10 12* triple mutant. Data from *shr phb arr7* and *pWOL:ARR10* provide further support for the role of PHB in B-ARR activities.

The behavior of PHB in the *shr* mutant was different to that reported previously for PHB in the cytokinin-signaling pathway. Our analysis of PHB in the protoplast assay suggests that PHB can suppress B-ARR activities. High cytokinin levels somehow augment the action of PHB on B-ARRs. The effect of cytokinin in this context might be related to changes in the phosphorylation status of B-ARRs, which somehow enhances repressive activities of PHB via physical interaction, competition for the same promoter element, or alteration of protein stability.

**Fig 8:**
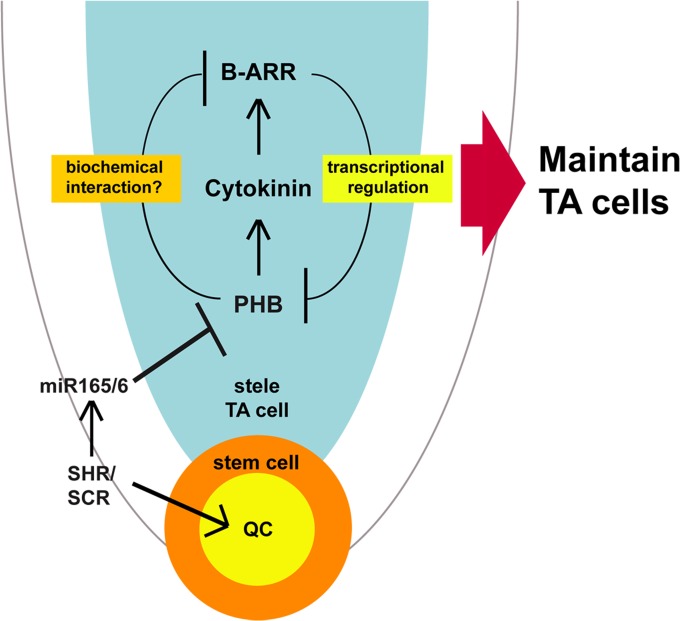
A model of SHR-PHB-cytokinin signaling in post-embryonic root growth. SHR/SCR posttranscriptionally regulates *PHB* expression in the stele via the *miRNA 165/6*. PHB in the stele promotes cytokinin biosynthesis, thereby enhancing B-ARR activities in one hand. On the other hand, PHB interferes with B-ARR activities depending on its concentration. Such regulation generates a negative feedback loop, which likely contributes to the homeostasis of cytokinin signaling.

On the basis of the results presented in this study, we propose a model explaining how the stem cell niche and TA cell activities are coordinated via the SHR, SCR, PHB, and cytokinin pathways (Fig. 8). In this model, SHR and SCR regulate root meristem and growth via two pathways. One pathway, which maintains the QC, is required for stem cell maintenance and the subsequent extension of root growth, and the other pathway, mediated by posttranscriptional suppression of *PHB*, is critical for TA cell proliferation. In the latter, PHB regulates the root meristem and growth activities by modulating local cytokinin production and B-ARR activities in a context dependent manner. Such a dynamic regulatory system might enable the root meristem to respond appropriately to the ever-changing growth environment. Future investigations into the detailed mechanisms underlying how PHB alters B-ARRs, could help to determine how these pathways orchestrate root meristem activity and growth.

## Methods

### Plant materials and treatment

The *Arabidopsis thaliana* mutant/marker lines used are listed in S4 Table. Ecotype Columbia, or lines introgressed into Columbia, were used in this study unless stated otherwise. Seeds were surface-sterilized and germinated on MS agar plates supplemented with 1% sucrose at 22–23°C under a photoperiod of 16-h light/8-h dark. The various mutant combinations used were generated via genetic crossing, and in each case, the genotypes were verified by polymerase chain reaction (PCR). The oligonucleotide sequences used are provided in S5 Table. For the suppressor screen, *shr* homozygous seeds were treated with EMS as previously described [64]. The *phb-15* and *phb-16* alleles were identified in reciprocal crosses with *shr-2 phb-6* mutants and subsequently confirmed by PCR sequencing. Both the *phb-15* and *16* alleles harbor a stop codon (C>>T) at amino acid positions 149 (exon 3) and 384 (exon 8), respectively.

### Histology, microscopy, RNA expression, and ChIP analysis

Primary roots of 5 to 21-day-old vertically grown seedlings were used for various analyses. Root lengths (from the base of the hypocotyl to the tip of the primary root) were measured using Image J software (www.rsbweb.nih.gov). The results presented here are the average data of 15–50 seedlings. GUS staining, Lugol staining, stereo (Leica DM5500B) and confocal microscopy (Leica TCS SP5 Laser Scanning Confocal Microscope) were conducted, with excitation (Ex) and emission (Em) wavelengths (Ex/Em) as follows: 405/461–504 nm for CFP, 488 nm/505–530 nm for GFP, 514 nm/525–555 nm for YFP, and 488–514 nm/613–679 nm for propidium iodide. Observation of callose on sieve plates in the phloem was performed as previously described [65]. In addition, qRT-PCR (using 2-mm-long segments of root tips, ABI 7900HT, Applied Biosystems), embryo analysis and whole-mount RNA *in situ* hybridization were performed as previously described [24], [66], [67], [68], [69], [70],. ChIP experiments (three biological replicates per experiment) were performed as previously described [7]. We used 5-day-old roots from seedlings expressing *pWOL:PHB-em:GFP*_*NLS*_. The oligonucleotide sequences used are listed in S5 Table.

### Microarray experiments

Wild-type, *shr* and *shr phb* plants harboring *pWOL:erGFP* were germinated and grown for 6 days on solid MS medium under the same conditions as described above. Apical half portions of roots were cut and harvested. Protoplasts were prepared from root tissues as previously described [32], and *pWOL:erGFP* expressing protoplasts were collected with an Aria high-speed flow cell sorter (BD Biosciences) and were subsequently used for microarray analysis. Total RNA was isolated from collected protoplasts using an RNeasy Plant Mini Kit (Qiagen). RNA integrity was determined using a bioanalyzer (Agilent BioAnalyzer 2100). Poly-adenylated RNA was isolated from total RNA using a RiboMinus Plant Kit for RNA-Seq (Invitrogen). Probe preparation was conducted according to the manufacturer’s instructions (GeneChip Whole Transcript Double-Stranded Target Assay Manual from Affymetrix Inc.), and then biotinylated double-stranded DNA probes were hybridized to the *Arabidopsis* Tiling 1.0R arrays (Affymetrix Inc.) at Cornell Life Sciences Core Laboratories Center. Two (*shr* and *shr phb* mutants) to three (wild-type plants) biological replicate datasets with high correlation coefficients were generated for analysis from each genetic line.

### Microarray data normalization and analysis

Microarray data were normalized using the RMA algorithm [71] in BIOCONDUCTOR, with Tiling 1.0R array CDF that contains gene-specific single-copy exonic probe sets [72]. Genes that were differentially expressed between the wild-type plant and the *shr* mutant were detected using the LIMMA package [73]. Principal component analysis (PCA) and clustering of differentially expressed genes were performed and visualized using MultiExperimental Viewer [74].

### Plasmids and constructs

*PHB-em*, generated via site-directed mutagenesis following the manufacturer’s protocol (QuikChange II Site-Directed Mutagenesis Kit, Stratagene), was cloned into pDONR221 (Invitrogen). The 4.5-kb upstream sequence of *WOX5* was PCR amplified from *Arabidopsis* genomic DNA and cloned into pDONRP4_P1R. *pWOX5:SHR:nlsGFP* was generated by fusing *SHR* cDNA to *nlsGFP* and the *WOX5* promoter. Transgenes were placed into dpGreen vectors using the Invitrogen MultiSite Gateway system and were mobilized into plants using the floral dip method, as previously described [75], [30]. For protoplast assays, we used a *pTCS:fLUC* (Firefly Luciferase) construct as a reporter system and a *pUBQ10:rLUC* (Renilla Luciferase) construct as an internal transfection control. For the effector construct, a region containing the coding sequences of target genes (*ARR10, PHB, PHB-em*) was inserted downstream of the 35S promoter using a modified pHBT-sGFP(S65T)-NOS vector. As empty vector, pSPYCE, a binary vector with cauliflower mosaic virus 35S promoter and the C-terminal (156–239 aa) domain of YFP, was used.

### Cytokinin measurement

Cytokinin quantification was performed as previously described [76], with some modifications. Briefly, ∼140 mg of the fresh half-bottom part of roots was collected from 6-day-old seedlings for each experimental replicate. Root tissues that were frozen in liquid nitrogen were processed in a vibration mill with tungsten carbide beads and 1-mL Bieleski buffer [77]. Deuterium-labeled cytokinin internal standards (Olchemim Ltd., Czech Republic) were added at the extraction stage, at 3 pmol per sample, to evaluate the recovery during purification and validate the quantification. The crude extract was then centrifuged (15 000 × *g* at 4°C), and the pellets were re-extracted in the same way. The combined supernatants were purified using a 1-g SCX column, evaporated to water phase in a vacuum followed by immunoaffinity chromatography using an immobilized wide-range anti-cytokinin monoclonal antibody as previously described [78]. The fraction containing cytokinin 9-glucosides, bases, and ribosides was evaporated to dryness, re-dissolved in the mobile phase and analyzed by UPLC-MS/MS [79]. The data were analyzed using Masslynx 4.1 software (Waters, Milford, MA, USA) and quantified by the standard isotope-dilution method.

### *Arabidopsis* protoplast transient expression: Reporter assay and real-time RT-PCR

*Arabidopsis* mesophyll protoplasts were isolated and transfected as previously described [80], and DNA was prepared using a large-scale preparation of high-quality plasmid DNA [81]. Protoplasts (6 × 10^4^ cells) were transfected with 40 μg of plasmid DNA with different combinations of reporter (*pTCS:fLUC*), effector (*p35S:ARR10, p35S:PHB, p35S:PHBem*), and internal control (*pUBQ:rLUC*) plasmids (S6 Table). Protoplasts were incubated in WI [0.5 M mannitol, 4 mM MES (pH 5.7), 20 mM KCl] at room temperature for 1 h in the light. After 1-h incubation, protoplasts were treated with 100 nM of 6-benzylaminopurine (BAP) and incubated for 5 h under the same conditions. Luciferase reporter activity was determined by the Dual Luciferase Assay System (Promega) and detected by Microlumat 96V Luminometer (Berthold, Bad Wildbad, Germany). To analyze *PHB* expression, quantitative RT-PCR analyses were carried out using total RNAs extracted from protoplasts (3 × 10^4^ cells), which were collected from protoplast transient assay. Total RNA extraction was performed with PicoPure RNA Isolation Kit (Arcturus). 20μL of reverse transcript reaction was performed for the first cDNA strand synthesis using 500ng of total RNAs and Superscript III reverse transcriptase (Invitrogen). For quantitative PCR reactions, a master mix was prepared using an iQ SYBR Green supermix (Bio-Rad) and PCR condition was programmed according to the manufacturer’s instructions for CFX96 Real-Time PCR machine (Bio-Rad). Three technical replicate reactions were performed. Primer information for RT-PCR is available in S5 Table. *Glyceraldehyde 3-phosphate dehydrogenase* (*GAPDH*) was used as internal control gene.

### Accession numbers

Microarray data have been submitted to the GEO database (accession number GSE33015). Sequence data from this article can be found in the *Arabidopsis* Genome Initiative or GenBank/EMBL databases under the following accession numbers: *SHR* (At4G37650), *PHB* (At2G34710), *PHV* (At1G30490), *CNA* (At1G52150), *ATHB8* (At4G32880), *REV* (At5G60690), *SCR* (At3G54220), *PLT1* (At3G20840), *PLT2* (At1G51190), *S32* (At2g18380), *AGL42* (At5g62165), *IPT3* (At3G63110), *IPT7* (At3G23630), *ARR7* (At1G19050), *ARR10* (At4G31920) and *GAPDH* (At3g26650).

## Supporting Information

S1 Fig Root meristem/growth recovery in *shr phb* and *scr phb* roots. (**A**) Restoration in root length in *shr phb* double mutants. New *phb* alleles; *phb-15* & *16* also resulted in a significant recovery in the *shr* root growth. (**B**) Root meristem size in wild-type, *shr-2, phb-6, shr-2 phb-6, scr-4*, and *scr-4 phb-6* plants (5 DAG). (**C**) Comparable levels of *pCycB1.2:GUS* expression in the proximal meristem cells in wild-type and *shr phb* roots (15 DAG). (**D**) Quantification of *pCycB1.2:GUS* expression is shown in the panel (**C**). White arrowheads marks the end of the meristem. Error bars represent the standard error (n = 15–30 plants). Scale bars: B, 50 μm; C, 20 μm.
(TIF)

S2 Fig Analysis of phloem sieve elements and QC in roots of the wild type, *shr*, and *shr phb*. (**A**) Aniline blue staining of sieve plates in phloem sieve elements in wild-type, *shr-2*, and *shr-2 phb-6* roots. Two sieve plates are organized in parallel in the wild-type root, whereas only one is found in the *shr-2* and *shr-2 phb-6* roots. (**B**) Expression patterns of known QC marker genes in wild-type, *shr-2* and *shr-2 phb-6* roots. Red arrowheads mark position of sieve plates and white arrowheads mark QC. Scale bars represent 25 μm.
(TIF)

S3 Fig *pWOX5:erGFP* expression during embryogenesis and precocious differentiation of columella stem cells in *shr phb* roots. (**A**) Similar levels of *pWOX5:erGFP* expression in wild-type, *shr-2*, and *shr-2 phb-6* embryos at the late heart stage. (**B**) A significant decrease in *pWOX5:erGFP* expression in *shr-2* and *shr-2 phb-6* embryos in contrast to the wild type at the bent-cotyledon stage. (**C**) Lugol’s staining shows precocious differentiation of columella stem cells in the *shr-2 phb-6* roots (5 DAG). Black and red arrowheads indicate QC and columella stem cells respectively. Scale bars represent A and B, 25 μm; C, 20 μm.
(TIF)

S4 Fig Increasing PHB levels in the stele of root meristem suppresses root growth in a QC-independent manner. (**A**) Four synonymous mutations introduced into the *miRNA 165/6* binding site of *PHB* via site-directed mutagenesis. Mutated bases are shown in purple. (**B)** A comparison of PHB-GFP expression domains in *pPHB:PHB:GFP, pPHB:PHB-m:GFP* and *pPHB:PHB:GFP shr-2* roots (5 DAG). Scale bars represent 20 μm. **(C**) A comparison of root meristem size in wild-type, *pWOL:PHB-m:GFP*_*NLS*_ and p*WOL:PHB-em:GFP*_*NLS*_ plants (upper panel: 7 DAG, lower panel: 5 DAG). Scale bars represent 20 μm (upper panel) and 10 μm (lower panel). White arrowheads mark where meristem ends. Asterisks indicate cortex cells. (**D**, **E**) Expression of *pWOX5:YFP* (**D**) and *pQC25:GUS* (**E**) in 15 day-old *pWOL:PHB-em:GFP*_*NLS*_ roots. (**F**) Aberrant cell divisions in the QC position of *pWOL:PHB-em:GFP*_*NLS*_ roots (10 DAG). (**G**) *WOX5 in situ* suggests decline in its expression levels in the QC region of *pWOL:PHB-em:GFP*_*NLS*_ roots. White arrows indicate QC cells undergoing abnormal cell divisions; Scale bars represent 25 μm for D-G.
(TIF)

S5 Fig *pWOL:erGFP* expression is stele-specific. Expression pattern of *pWOL:erGFP* in the wild-type, *shr-2*, and *shr-2 phb-6* roots. Scale bars represent 25 μm.
(TIF)

S6 Fig Genome-wide expression data from the stele cells of wild-type, *shr*. and *shr phb* roots. (**A**) Correlation coefficients (R2) between cell sorting/tiling array data. Biological replicates are highlighted in yellow. (**B**, **C**) Expression dynamics of differentially expressed genes between the *shr* and wild-type plants, summarized with PCA (**B**) and QT clustering (**C**). *shr-2*, SHR1 and 2; wild type, WT1, 2 and 3; *shr-2 phb-6*, DM1 and 2.
(TIF)

S7 Fig Cell type-specific expression patterns in the *Arabidopsis* roots for genes that are repressed (A) and activated (B) by PHB. Expression patterns are classified by Hierarchical Clustering.
(TIF)

S8 Fig SHR-PHB pathway and auxin signaling. (**A**) *pDR5:YFPvenus* shows a reduced signal in *shr-2* mutants in comparison to wild-type and *shr-2 phb-6* plants (7 DAG). (**B**, **C**) Reduced expression of *pPIN1:PIN1:GFP* in 5- (**B**) and 10- (**C**) day-old *shr-2* roots in comparison to the wild type. Expression is partially restored in *shr-2 phb-6*. Scale bars represent 25 μm.
(TIF)

S9 Fig Changes in A-*ARR* expression correlate with the enhanced cytokinin responsiveness in the *shr* root. (**A**) Cytokinin content in the wild-type, *shr-2* and *shr-2 phb-6* roots. tZ, Transzeatin; tZR, tZ riboside; cZ, cis-zeatin; cZR, cZ riboside; iP, N6-(Δ2-isopentenyl) adenine; iPR, iP riboside; DHZ, Dihydrozeatin; DHZR, Dihydrozeatin riboside; DHZ9G, DHZ-9-glucoside; iP9G, iP-9-glucoside; tZ9G, tZ-9-glucoside; cZ9G, cZ-9-glucoside. (**B**) Heat map showing expression levels of A-*ARR*s in the wild-type (WT1, 2 and 3), *shr* (SHR1 and 2), and *shr phb* (DM1 and 2) root stele. (**C**) Relative mRNA levels of *ARR5, ARR7* and *ARR15* in the *shr-2* and *shr-2 phb-6* roots. Data are normalized to the wild-type plants. (**D**) Root growth in *shr-2 phb-6 arr7* mutant. (**E**) Expression pattern of *pTCS:erGFP* in *phb-7d* roots. (**F**) *pS32:erGFP* expression in the wild-type, *shr-2* and *shr-2 phb-6* roots. Scale bars represent 25 μm. SD: standard deviation (n = 3 biological replicates).
(TIF)

S10 Fig Changes in *pTCS:erGFP* expression under the inducible PHB-em (**A**) Quantitative comparison of TCS-GFP levels in the stele and root cap expressing the wild type (WT) and transgenic line expressing PHB-em fused to the glucocorticoid receptor (GR) under the PHB promoter. (**B**) Expression patterns of *pTCS:erGFP* in WT and transgenic line crossed with *pPHB:PHBem:GR*. All seedlings grew under the same conditions. After 5 DAG, seedlings were transferred to new media including no DEX or 10 uM DEX and were incubated for 3 h. GFP expression is reduced following treatment with 10 uM DEX in transgenic lines.
(TIF)

S11 Fig PHB suppresses B-ARR activities in the presence of high cytokinin in a concentration-dependent manner. **(A)** A high dosage of PHB (*p35S:PHB-em*) suppressed ARR10 activities under the high cytokinin. In contrast, PHB expressed by *p35S:PHB* was not effective on ARR10 activities. (**B**) *PHB* expression is analyzed by qRT-PCR using 100 nM BAP-treated protoplasts. The error bar represents standard deviation. BAP, 6-benzylaminopurine.
(TIF)

S1 Table Normalized gene expression data in *shr-2* (SHR1 and 2), wild-type (WT1, 2 and 3) and *shr-2 phb-6* (DM1 and 2) plants. (XLS)

S2 Table Genes that are differentially expressed in the stele cells of *shr*-2 and wild-type roots, classified by QT clustering. (XLS)

S3 Table Over-representation of biological functions by genes that are activated or repressed by high PHB concentrations in *shr-2* mutants. (XLS)

S4 Table Details of *Arabidopsis* mutant/marker lines used in this study. (XLS)

S5 Table Primer sequences used for cloning, genotyping, sequencing, qRT-PCR, RNA in situ hybridization, and ChIP-PCR. (XLS)

S6 Table A combination of gene constructs transfected into *Arabidopsis* leaf mesophyll protoplasts. (XLSX)
