# Supplementary figures and images for: PHABULOSA Controls the Quiescent Center-Independent Root Meristem Activities in Arabidopsis thaliana
Source: PLoS Genet. 2015 Mar 2;11(3):e1004973. doi: 10.1371/journal.pgen.1004973 (PMC4346583; doi:10.1371/journal.pgen.1004973)

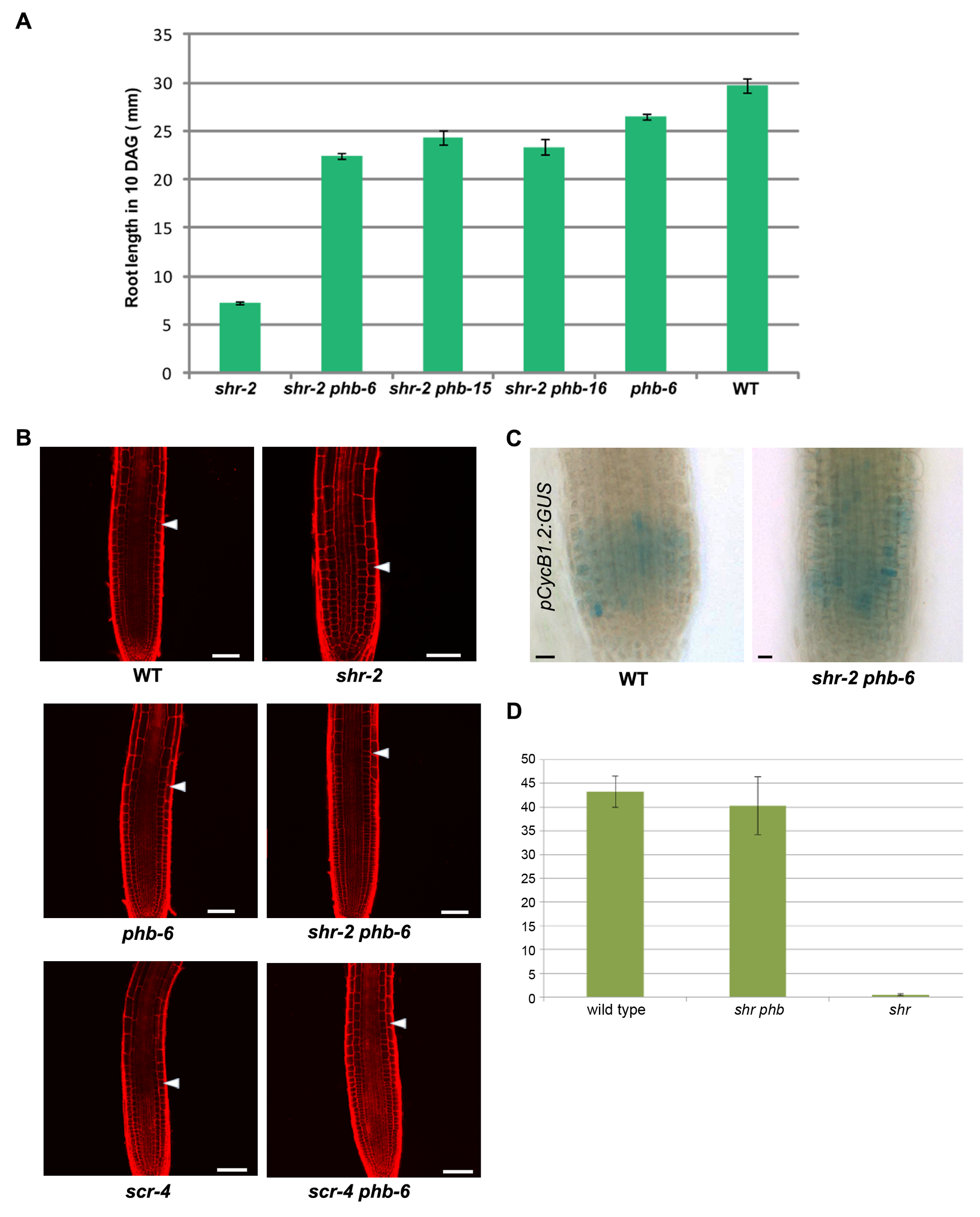

Supplement: S1 Fig — (A) Restoration in root length in shr phb double mutants. New phb alleles; phb-15 & 16 also resulted in a significant recovery in the shr root growth. (B) Root meristem size in wild-type, shr-2, phb-6, shr-2 phb-6, scr-4, and scr-4 phb-6 plants (5 DAG). (C) Comparable levels of pCycB1.2:GUS expression in the proximal meristem cells in wild-type and shr phb roots (15 DAG). (D) Quantification of pCycB1.2:GUS expression is shown in the panel (C). White arrowheads marks the end of the meristem. Error bars represent the standard error (n = 15–30 plants). Scale bars: B, 50 μm; C, 20 μm. (TIF) [file pgen.1004973.s001.tif]

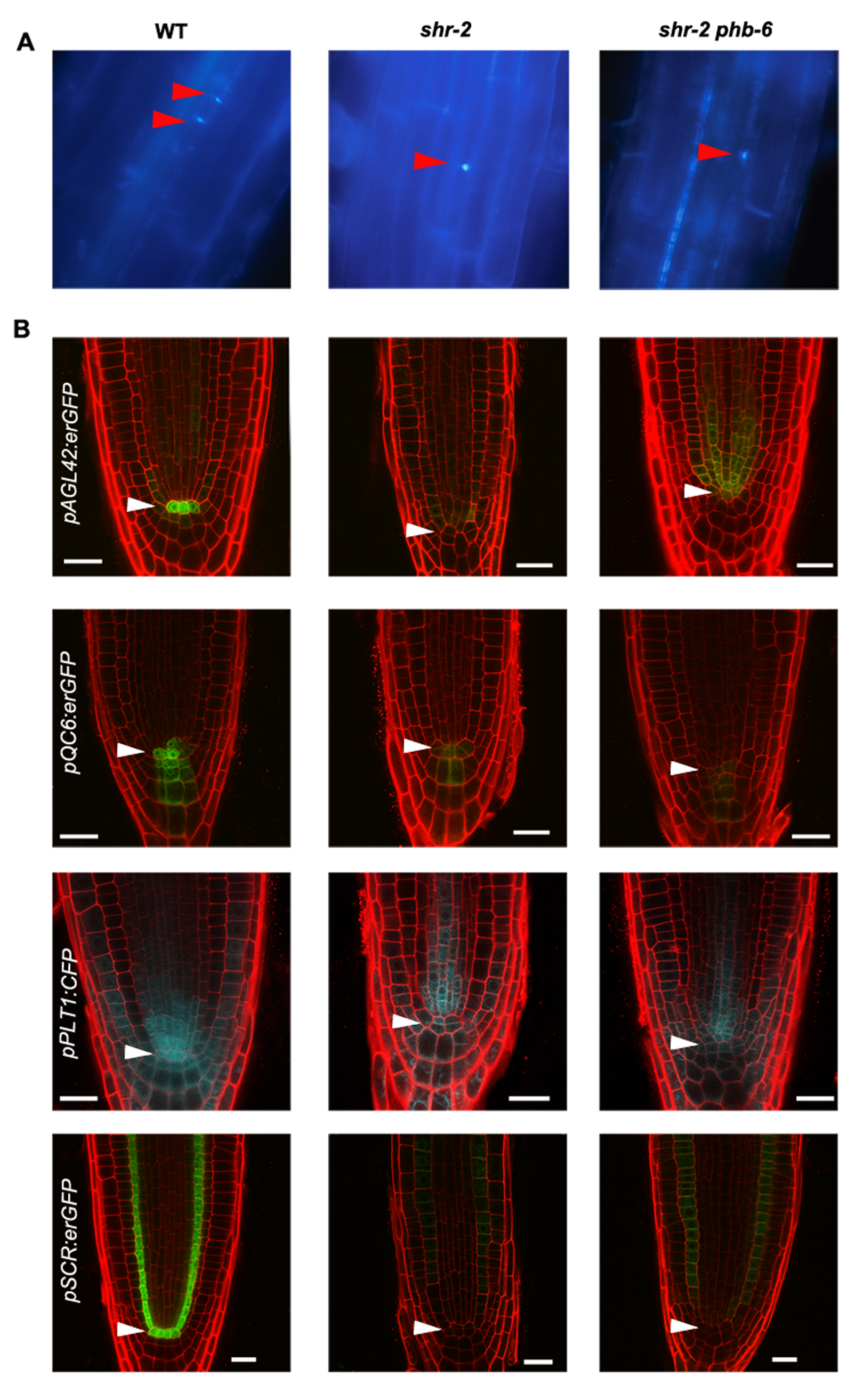

Supplement: S2 Fig — (A) Aniline blue staining of sieve plates in phloem sieve elements in wild-type, shr-2, and shr-2 phb-6 roots. Two sieve plates are organized in parallel in the wild-type root, whereas only one is found in the shr-2 and shr-2 phb-6 roots. (B) Expression patterns of known QC marker genes in wild-type, shr-2 and shr-2 phb-6 roots. Red arrowheads mark position of sieve plates and white arrowheads mark QC. Scale bars represent 25 μm. (TIF) [file pgen.1004973.s002.tif]

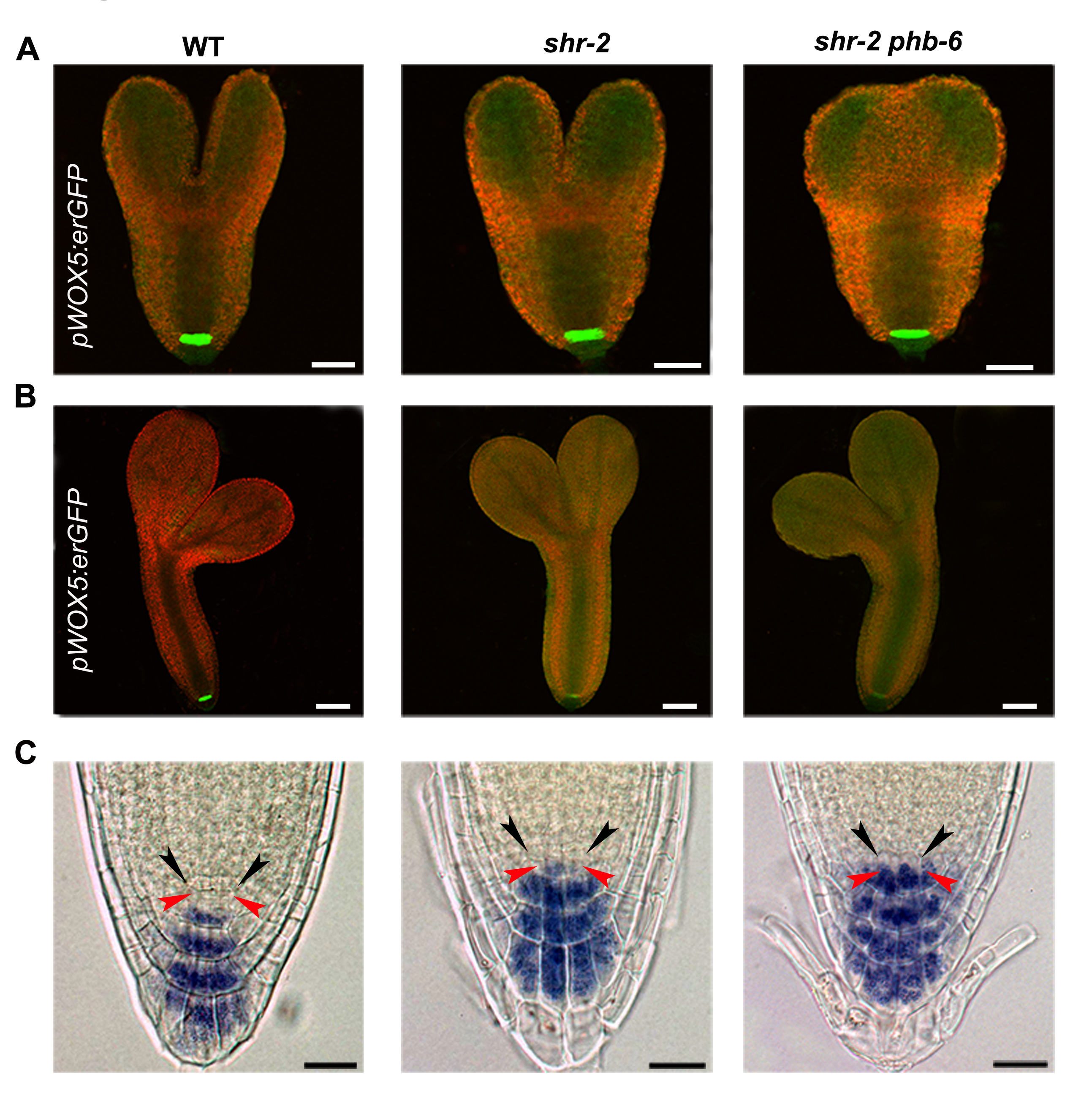

Supplement: S3 Fig — (A) Similar levels of pWOX5:erGFP expression in wild-type, shr-2, and shr-2 phb-6 embryos at the late heart stage. (B) A significant decrease in pWOX5:erGFP expression in shr-2 and shr-2 phb-6 embryos in contrast to the wild type at the bent-cotyledon stage. (C) Lugol’s staining shows precocious differentiation of columella stem cells in the shr-2 phb-6 roots (5 DAG). Black and red arrowheads indicate QC and columella stem cells respectively. Scale bars represent A and B, 25 μm; C, 20 μm. (TIF) [file pgen.1004973.s003.tif]

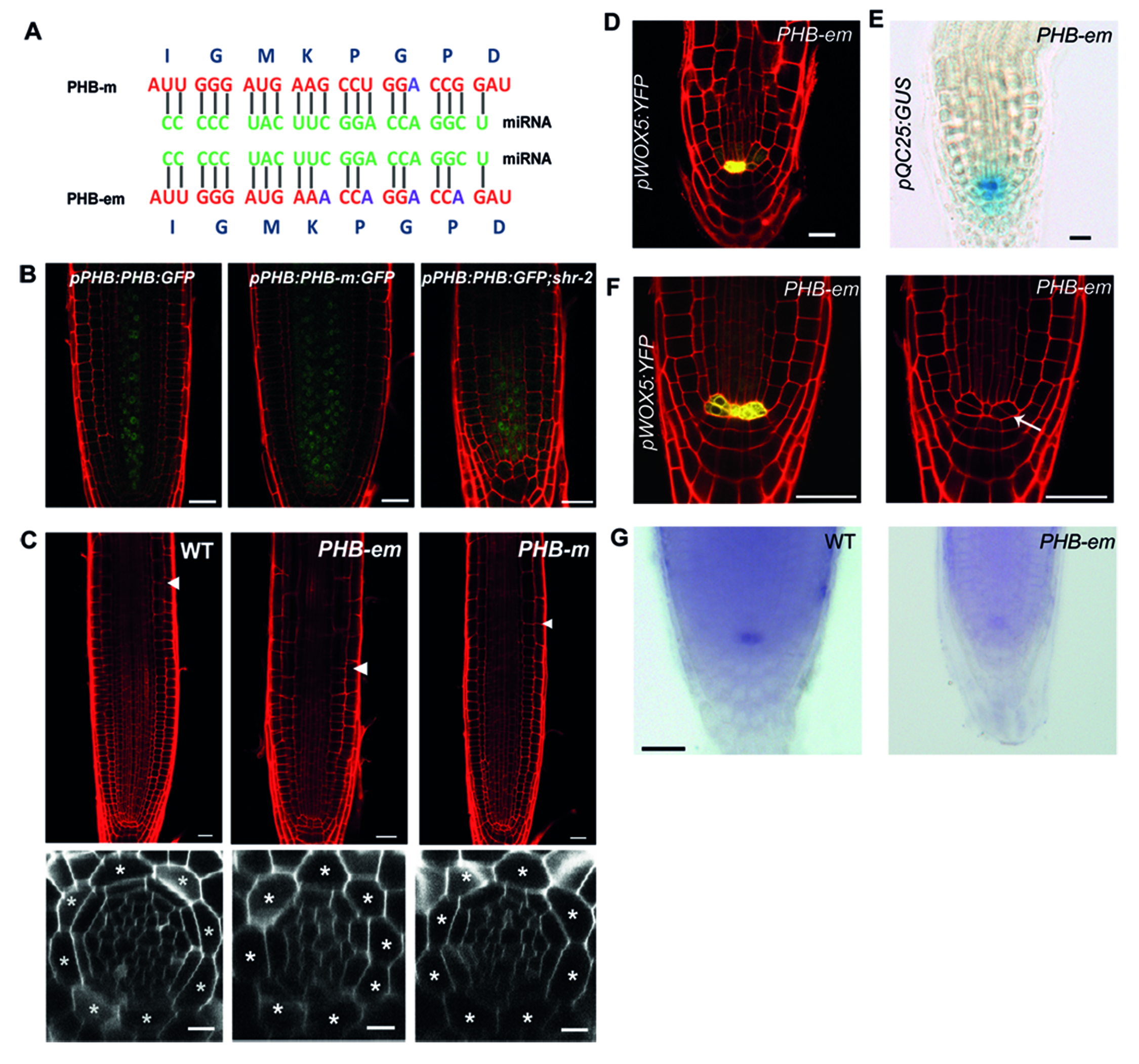

Supplement: S4 Fig — (A) Four synonymous mutations introduced into the miRNA 165/6 binding site of PHB via site-directed mutagenesis. Mutated bases are shown in purple. (B) A comparison of PHB-GFP expression domains in pPHB:PHB:GFP, pPHB:PHB-m:GFP and pPHB:PHB:GFP shr-2 roots (5 DAG). Scale bars represent 20 μm. (C) A comparison of root meristem size in wild-type, pWOL:PHB-m:GFP NLS and pWOL:PHB-em:GFP NLS plants (upper panel: 7 DAG, lower panel: 5 DAG). Scale bars represent 20 μm (upper panel) and 10 μm (lower panel). White arrowheads mark where meristem ends. Asterisks indicate cortex cells. (D, E) Expression of pWOX5:YFP (D) and pQC25:GUS (E) in 15 day-old pWOL:PHB-em:GFP NLS roots. (F) Aberrant cell divisions in the QC position of pWOL:PHB-em:GFP NLS roots (10 DAG). (G) WOX5 in situ suggests decline in its expression levels in the QC region of pWOL:PHB-em:GFP NLS roots. White arrows indicate QC cells undergoing abnormal cell divisions; Scale bars represent 25 μm for D-G. (TIF) [file pgen.1004973.s004.tif]

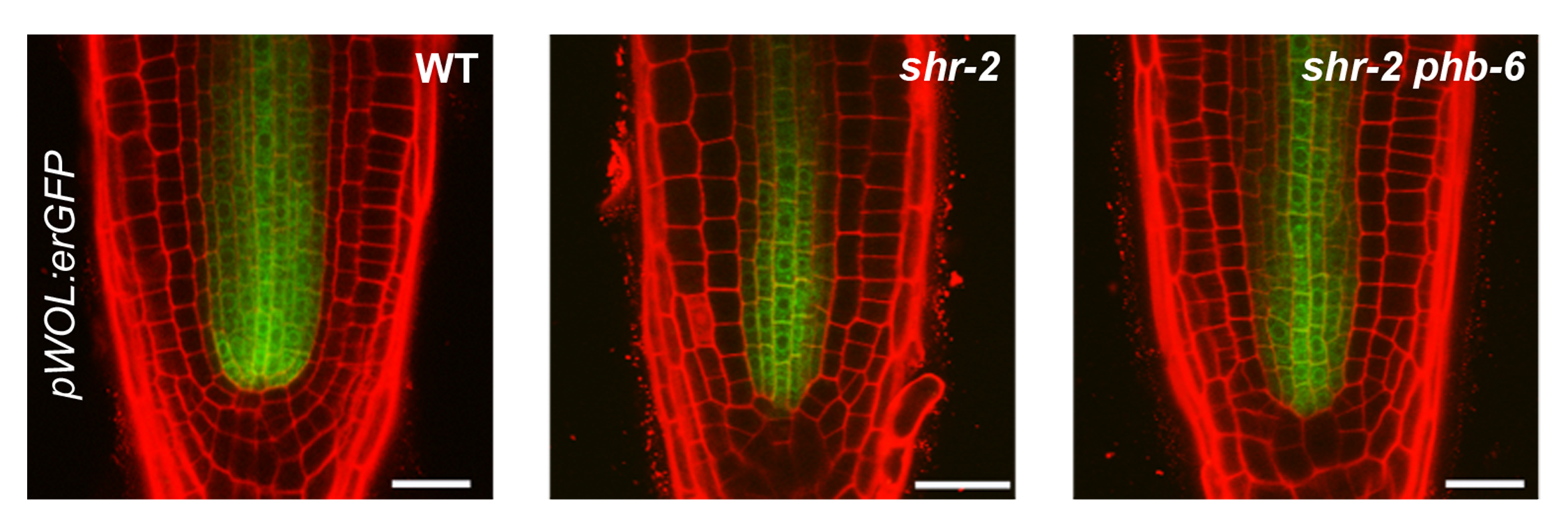

Supplement: S5 Fig — Expression pattern of pWOL:erGFP in the wild-type, shr-2, and shr-2 phb-6 roots. Scale bars represent 25 μm. (TIF) [file pgen.1004973.s005.tif]

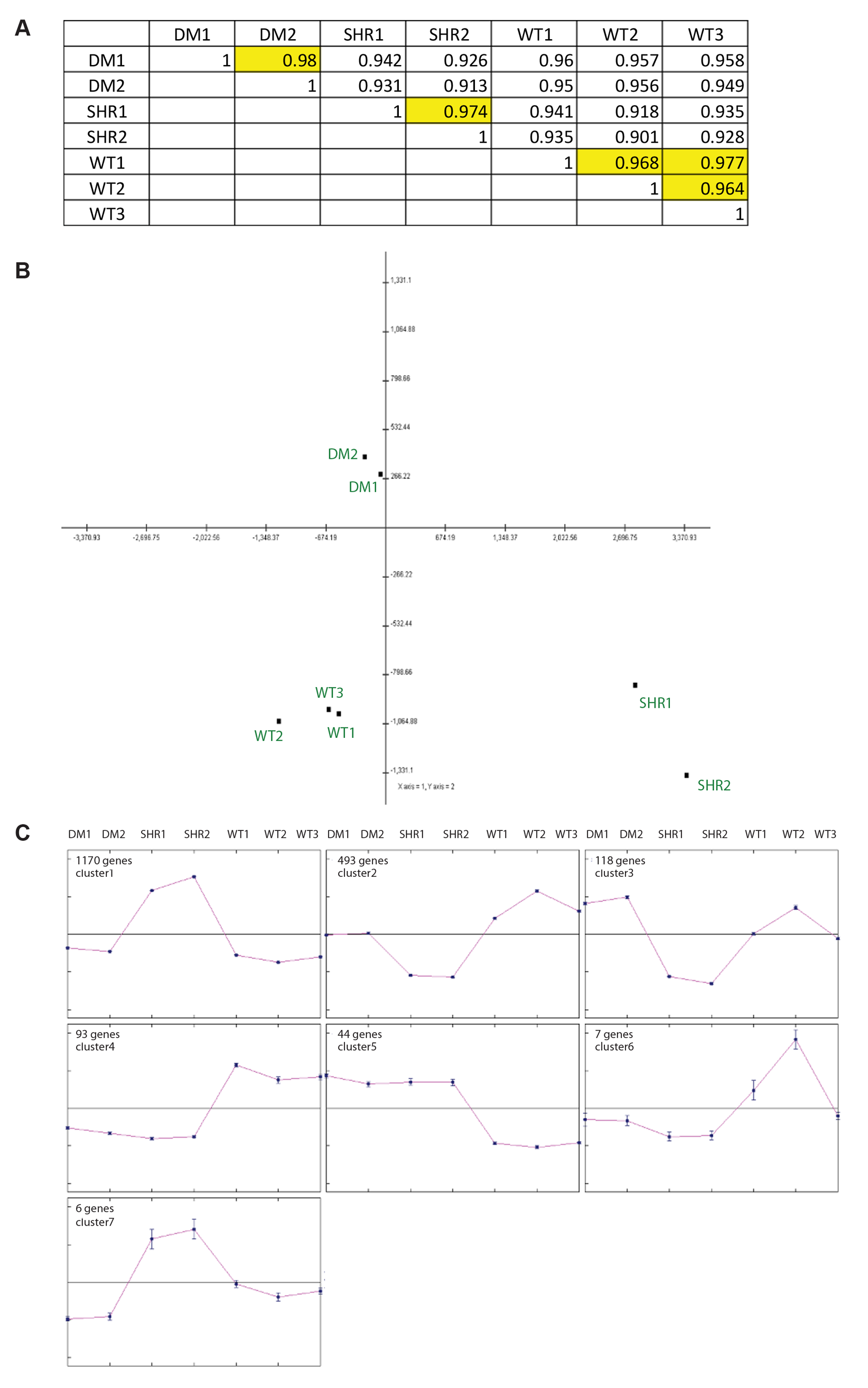

Supplement: S6 Fig — (A) Correlation coefficients (R2) between cell sorting/tiling array data. Biological replicates are highlighted in yellow. (B, C) Expression dynamics of differentially expressed genes between the shr and wild-type plants, summarized with PCA (B) and QT clustering (C). shr-2, SHR1 and 2; wild type, WT1, 2 and 3; shr-2 phb-6, DM1 and 2. (TIF) [file pgen.1004973.s006.tif]

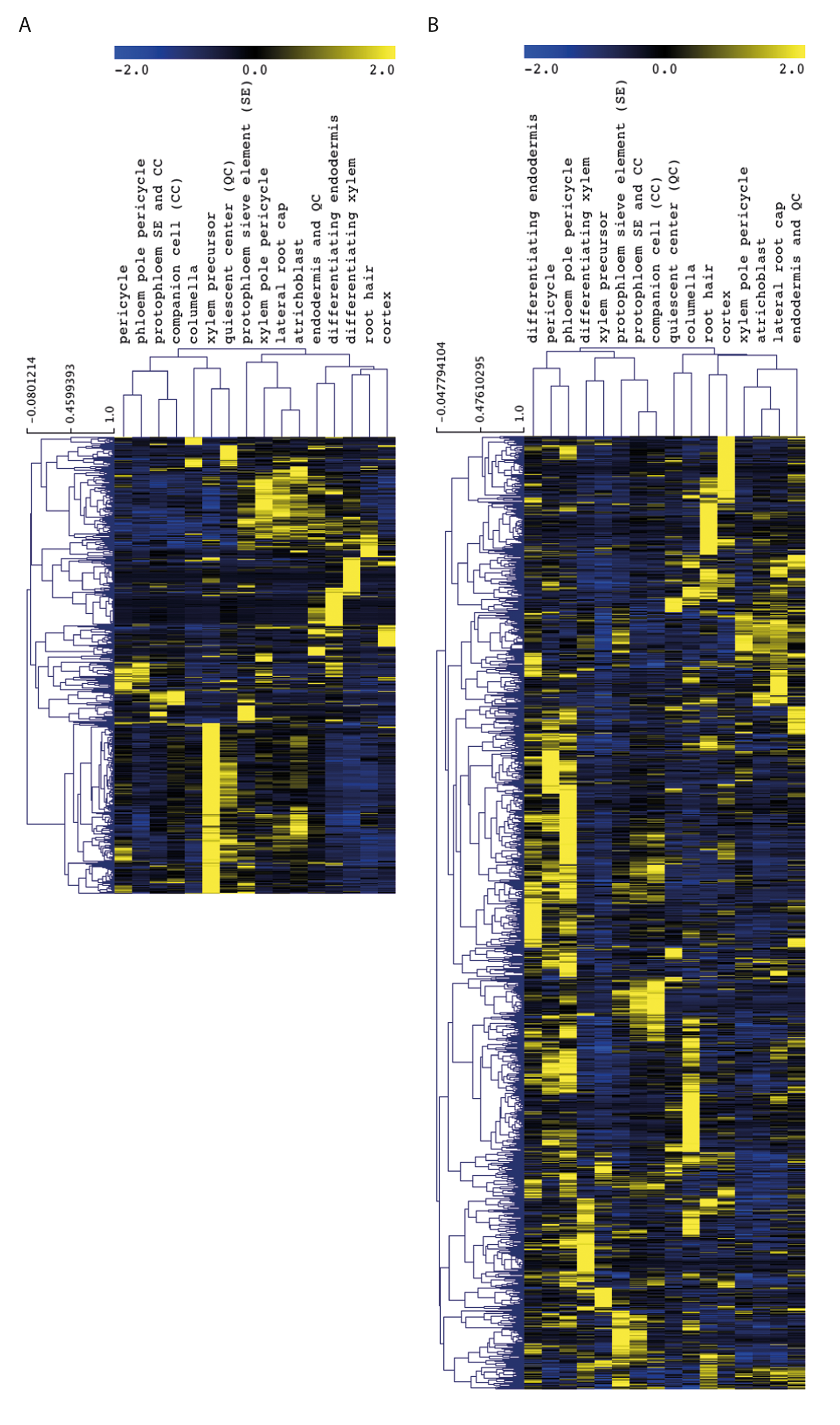

Supplement: S7 Fig — Expression patterns are classified by Hierarchical Clustering. (TIF) [file pgen.1004973.s007.tif]

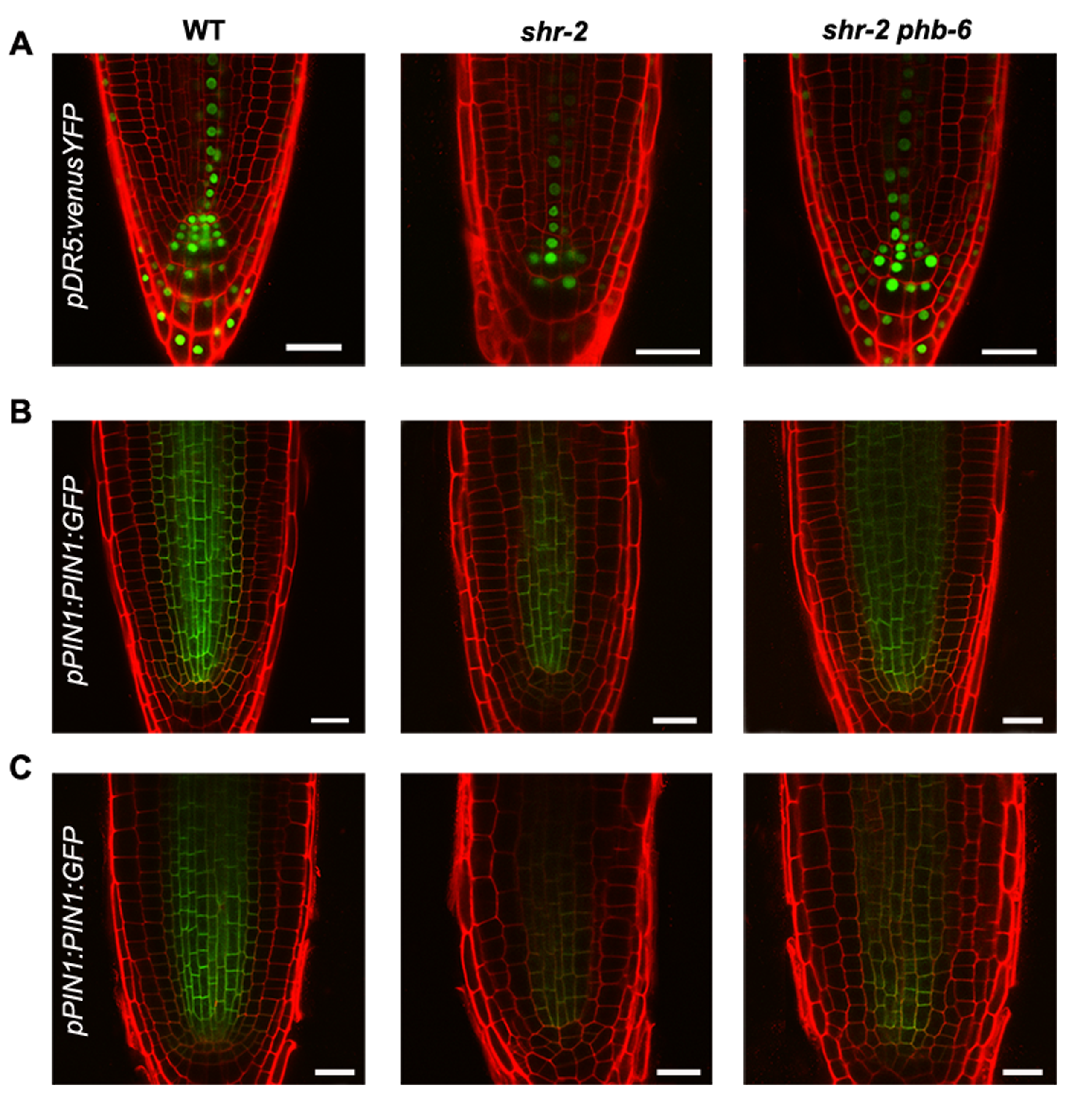

Supplement: S8 Fig — (A) pDR5:YFPvenus shows a reduced signal in shr-2 mutants in comparison to wild-type and shr-2 phb-6 plants (7 DAG). (B, C) Reduced expression of pPIN1:PIN1:GFP in 5- (B) and 10- (C) day-old shr-2 roots in comparison to the wild type. Expression is partially restored in shr-2 phb-6. Scale bars represent 25 μm. (TIF) [file pgen.1004973.s008.tif]

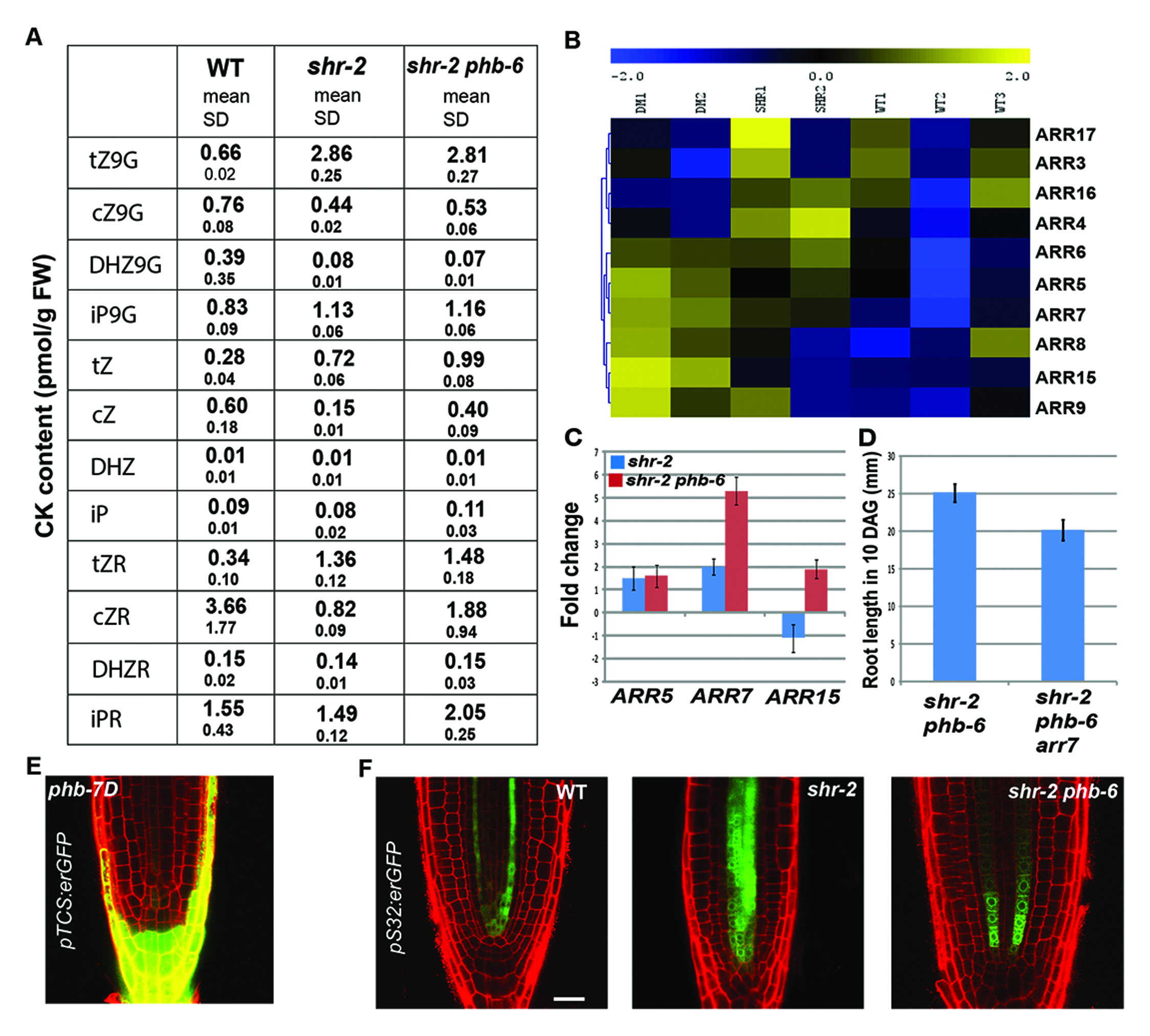

Supplement: S9 Fig — (A) Cytokinin content in the wild-type, shr-2 and shr-2 phb-6 roots. tZ, Transzeatin; tZR, tZ riboside; cZ, cis-zeatin; cZR, cZ riboside; iP, N6-(Δ2-isopentenyl) adenine; iPR, iP riboside; DHZ, Dihydrozeatin; DHZR, Dihydrozeatin riboside; DHZ9G, DHZ-9-glucoside; iP9G, iP-9-glucoside; tZ9G, tZ-9-glucoside; cZ9G, cZ-9-glucoside. (B) Heat map showing expression levels of A-ARRs in the wild-type (WT1, 2 and 3), shr (SHR1 and 2), and shr phb (DM1 and 2) root stele. (C) Relative mRNA levels of ARR5, ARR7 and ARR15 in the shr-2 and shr-2 phb-6 roots. Data are normalized to the wild-type plants. (D) Root growth in shr-2 phb-6 arr7 mutant. (E) Expression pattern of pTCS:erGFP in phb-7d roots. (F) pS32:erGFP expression in the wild-type, shr-2 and shr-2 phb-6 roots. Scale bars represent 25 μm. SD: standard deviation (n = 3 biological replicates). (TIF) [file pgen.1004973.s009.tif]

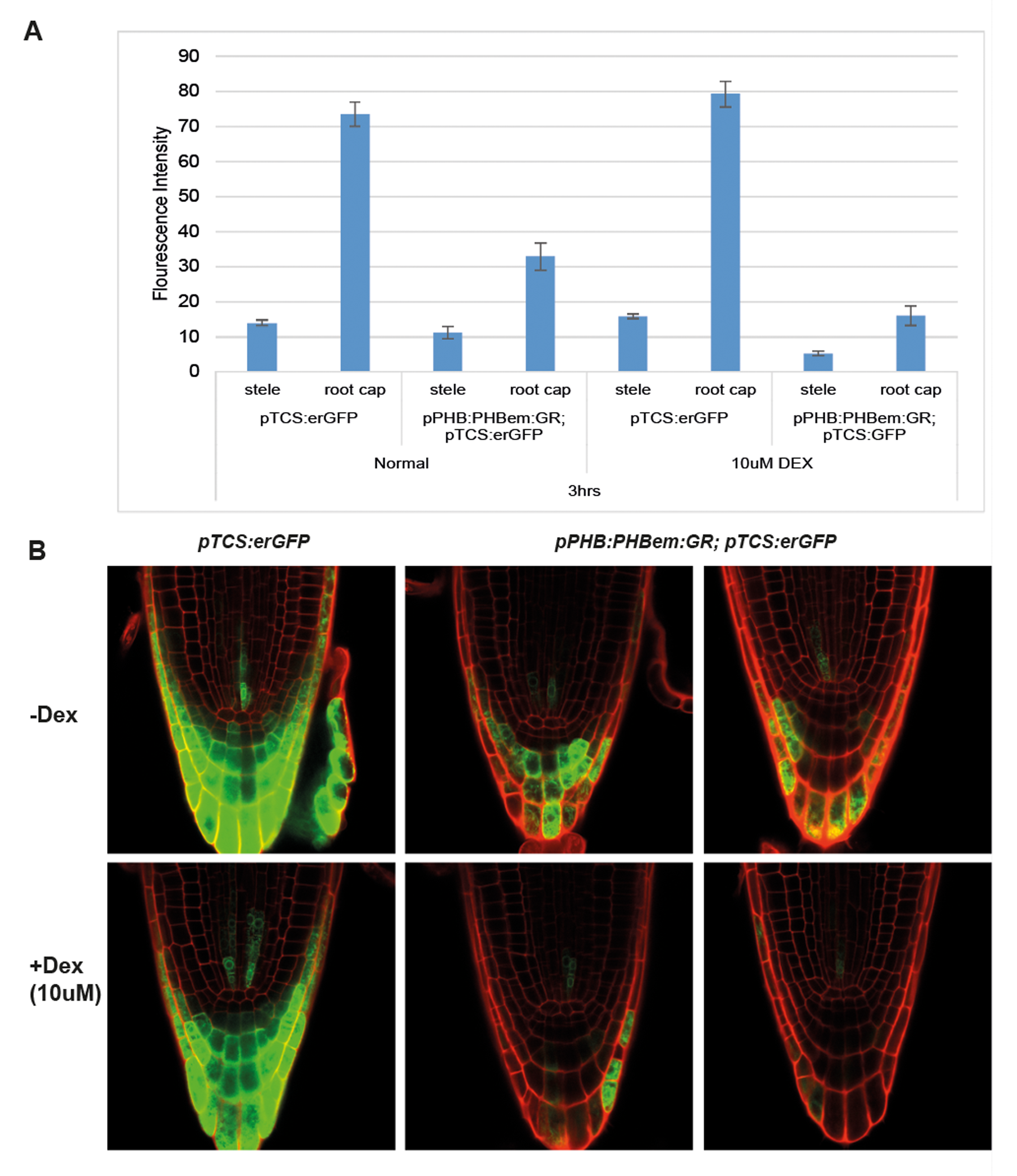

Supplement: S10 Fig — (A) Quantitative comparison of TCS-GFP levels in the stele and root cap expressing the wild type (WT) and transgenic line expressing PHB-em fused to the glucocorticoid receptor (GR) under the PHB promoter. (B) Expression patterns of pTCS:erGFP in WT and transgenic line crossed with pPHB:PHBem:GR. All seedlings grew under the same conditions. After 5 DAG, seedlings were transferred to new media including no DEX or 10 uM DEX and were incubated for 3 h. GFP expression is reduced following treatment with 10 uM DEX in transgenic lines. (TIF) [file pgen.1004973.s010.tif]

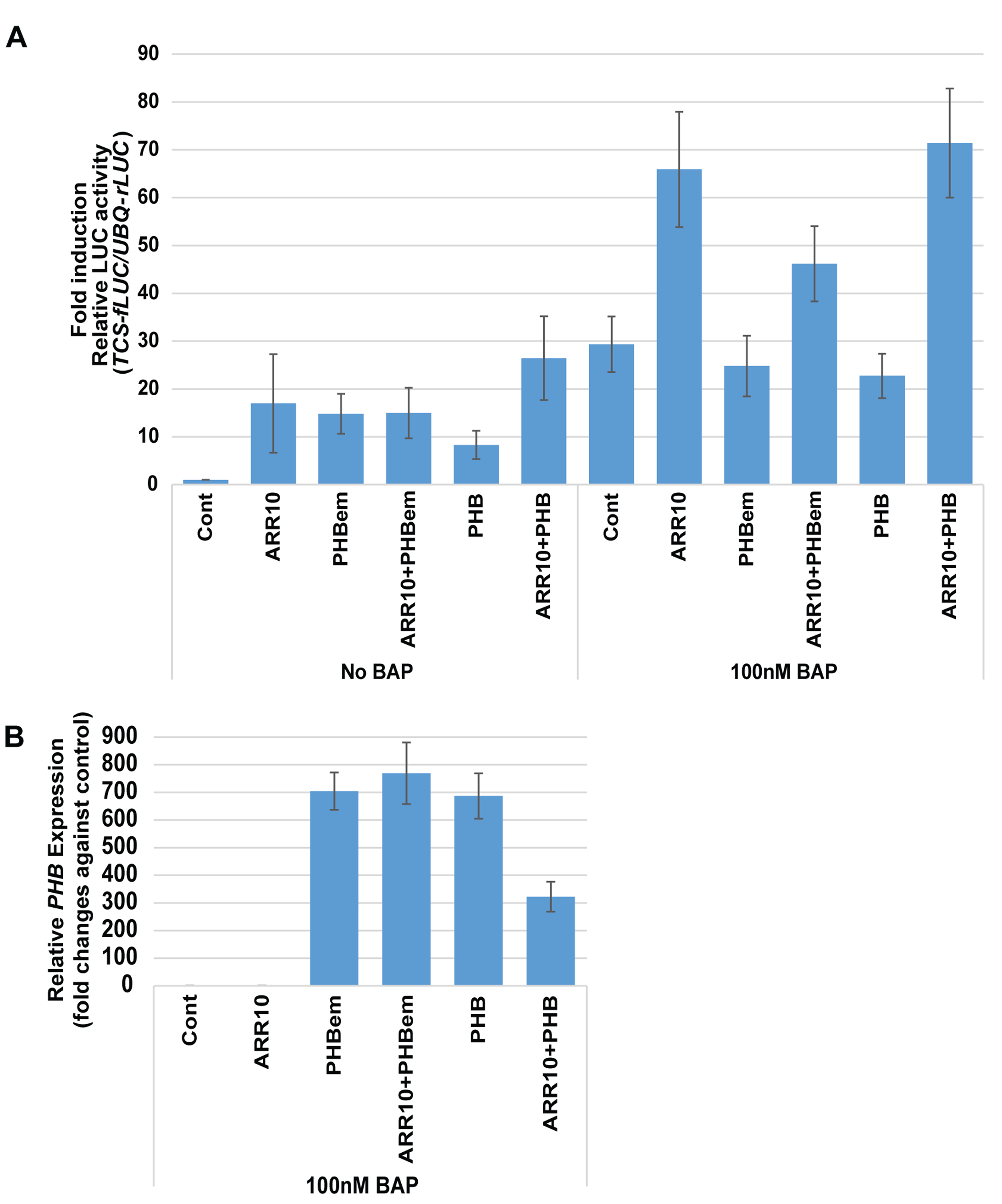

Supplement: S11 Fig — (A) A high dosage of PHB (p35S:PHB-em) suppressed ARR10 activities under the high cytokinin. In contrast, PHB expressed by p35S:PHB was not effective on ARR10 activities. (B) PHB expression is analyzed by qRT-PCR using 100 nM BAP-treated protoplasts. The error bar represents standard deviation. BAP, 6-benzylaminopurine. (TIF) [file pgen.1004973.s011.tif]
